# An Experimental Approach to Assess Fluorine Incorporation into Disordered Rock Salt Oxide Cathodes

**DOI:** 10.1021/acs.chemmater.3c03138

**Published:** 2024-04-03

**Authors:** Raynald Giovine, Eric Yoshida, Vincent C. Wu, Yuefan Ji, Matthew J. Crafton, Bryan D. McCloskey, Raphaële J. Clément

**Affiliations:** † Materials Department, University of California, Santa Barbara, Santa Barbara, California 93106, United States; ‡ Materials Research Laboratory, University of California, Santa Barbara, Santa Barbara, California 93106, United States; § Department of Chemical and Biomolecular Engineering, University of California, Berkeley, Berkeley, California 94720, United States; ∥ Energy Storage and Distributed Resources Division, Lawrence Berkeley National Laboratory, Berkeley, California 94720, United States

## Abstract

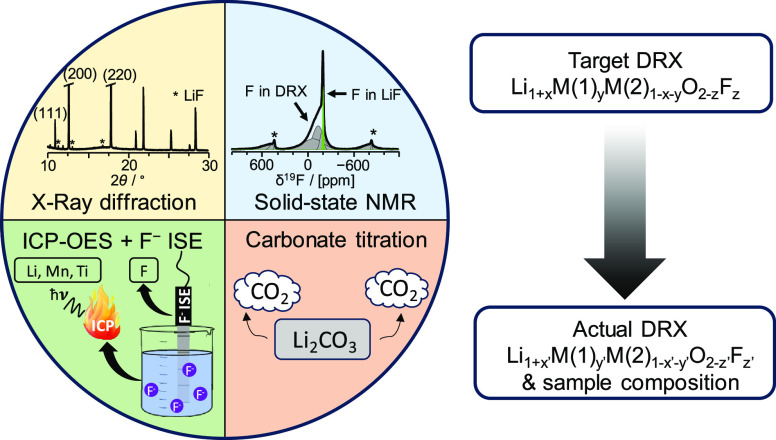

Disordered rock salt oxides (DRX) have shown great promise as high-energy-density and sustainable Li-ion cathodes. While partial substitution of oxygen for fluorine in the rock salt framework has been related to increased capacity, lower charge–discharge hysteresis, and longer cycle life, fluorination is poorly characterized and controlled. This work presents a multistep method aimed at assessing fluorine incorporation into DRX cathodes, a challenging task due to the difficulty in distinguishing oxygen from fluorine using X-ray and neutron-based techniques and the presence of partially amorphous impurities in all DRX samples. This method is applied to “Li_1.25_Mn_0.25_Ti_0.5_O_1.75_F_0.25_” prepared by solid-state synthesis and reveals that the presence of LiF impurities in the sample and F content in the DRX phase is well below the target. Those results are used for compositional optimization, and a synthesis product with drastically reduced LiF content and a DRX stoichiometry close to the new target composition (Li_1.25_Mn_0.225_Ti_0.525_O_1.85_F_0.15_) is obtained, demonstrating the effectiveness of the strategy. The analytical method is also applied to “Li_1.33_Mn_0.33_Ti_0.33_O_1.33_F_0.66_” obtained via mechanochemical synthesis, and the results confirm that much higher fluorination levels can be achieved via ball-milling. Finally, a simple and rapid water washing procedure is developed to reduce the impurity content in as-prepared DRX samples: this procedure results in a ca. 10% increase in initial discharge capacity and a ca. 11% increase in capacity retention after 25 cycles for Li_1.25_Mn_0.25_Ti_0.50_O_1.75_F_0.25_. Overall, this work establishes new analytical and material processing methods that enable the development of more robust design rules for high-energy-density DRX cathodes.

## Introduction

Decarbonization of transportation and energy sources relies on the massive deployment of energy storage technologies. While pumped-storage hydropower is still the most widely deployed grid-scale storage technology, with a total installed capacity of around 160 GW in 2021, grid-scale batteries are catching up: the total grid-scale battery storage capacity stood close to 16 GW at the end of 2021, most of which was added over the course of the previous 5 years.^1^ In addition, automotive lithium-ion battery (LIB) demand has increased by about 65% to 550 GWh in 2022.^2^ Importantly, the surge in LIB demand drives the demand for critical minerals, including Co and Ni, that make up most of the cathode materials (e.g., LiCoO_2_, Li(Ni,Mn,Co)O_2_ (NMC), and Li(Ni,Co,Al) (NCA)) commercialized today.^3−5^ These oxide cathodes with a layered rock salt structure exhibit high energy densities (up to 760 Wh/kg for NMC811)^6−12^ unmatched by current, more sustainable cathode chemistries, e.g., LiFePO_4_ (up to 544 Wh/kg).^6−8^ Yet, a new class of oxide cathodes with a cation-disordered rock salt (DRX) structure has garnered significant interest over the past few years due to their extremely high energy densities (>900 Wh/kg)^9,10^ and the ability to leverage Earth-abundant redox-active metals (e.g., Mn^2+/4+^).^10,11^

In the DRX structure, Li and transition metals (TMs) no longer occupy separate layers as in traditional oxide cathodes and are instead quasi-randomly distributed on the cation lattice. While a limited subset of layered transition-metal oxides are structurally stable during electrochemical cycling (those containing redox-active Co^3+^ and Ni^2+/3+^ and redox-inactive Mn^4+^), cation disorder in DRX opens a much larger compositional space for exploration.^9^ Among possible redox-active metals, Mn has attracted particular interest as it can exchange up to two electrons (going from Mn^2+^ to Mn^4+^) and is widely distributed on the Earth’s crust and therefore readily available and low cost.^10−18^ DRX cathodes are typically prepared with ≥10% Li excess (Li stoichiometry ≥1.1) to enable long-range Li^+^ diffusion through the structure. Similarly to layered oxide cathodes, long-range Li^+^ transport involves hops between adjacent octahedral Li sites via a tetrahedral transition site (so-called *O*–*T*–*O* diffusion pathways), but in the case of DRX, this process mostly involves Li-rich environments or 0-TM channels (i.e., tetrahedral transition sites with no TM species in face-sharing octahedral sites) that must form a three-dimensional (3D) percolating network for long-range Li^+^ transport. Most DRX cathodes also contain TM species with no electrons in their d shell (d^0^) that impart stability as they can accommodate the highly distorted octahedral sites present in the structure.^9^ Another important difference between layered and disordered oxides is the amenability of the latter to fluorine substitution,^19^ whereas attempts to fluorinate layered oxides have all resulted in the formation of a separate LiF phase.^20,21^ The fluorination of the DRX oxides presents several advantages. On the one hand, F^–^ anions lower the average anion valence, enabling the incorporation of a greater fraction of low-valent (e.g., Li^+^ or redox-active Mn^2+^) metals on the cation lattice, which in turn increases the more reversible, TM-based redox capacity and reduces the need for poorly reversible and hysteretic anion-based redox.^15,22^ On the other hand, fluorination has been found to enhance the surface stability of DRX cathodes during cycling.^22^ Finally, several studies have highlighted the impact of fluorination on cation short-range order (SRO) and therefore Li^+^ transport.^15,23−25^

Given the complex interplay among composition, SRO, and electrochemical properties, it has become clear that further improvements in DRX performance, such as reduced capacity fade, reduced voltage hysteresis, and enhanced power capability, necessitate precise control over their Li and F stoichiometry. Yet, quantifying the amount of F incorporated into the bulk DRX lattice has proven challenging. Notably, neither X-rays nor neutrons can distinguish O from F, and most studies still report the target DRX stoichiometry (based on the ratio of precursors used in the synthesis) or the Li/TM/O elemental ratio in the sample rather than the actual stoichiometry of the DRX phase (including F). Consequently, DRX material design rules remain elusive, slowing their large-scale adoption.

The present work establishes a broadly applicable experimental methodology to assess F incorporation into DRX cathodes. The procedure combines long-range structural characterization (X-ray diffraction (XRD)) and local structure probes (^7^Li and ^19^F solid-state NMR (ss-NMR)) to assess the purity of the sample. Solid-state NMR is a particularly valuable tool for the characterization of DRX materials, as it is sensitive to both crystalline and amorphous phases in the sample, and ^19^F ss-NMR allows one to directly probe the distribution of local F environments in the DRX phase and in potential impurities. Those structural tools are complemented with compositional analyses, including inductively coupled plasma optical emission spectroscopy (ICP-OES), fluoride ion-selective electrode (F-ISE) measurements, and carbonate titration. We apply this methodology to Li–Mn^2+^–Ti^4+^–O–F DRX compounds prepared via standard solid-state and mechanochemical milling synthesis, which have shown significant promise as high-energy-density cathodes. The initial focus is on the Li_1.25_Mn_0.25_Ti_0.50_O_1.75_F_0.25_ composition (denoted as LMTF25) prepared via solid-state synthesis. The targeted F content is higher than the expected F solubility limit,^25^ and we indeed find that a significant fraction of the F in the pristine DRX sample forms LiF impurities instead of being incorporated into the cathode structure, while the high-temperature calcination step leads to significant Li and F losses. Based on those results, we devised a new DRX composition with a F content closer to the observed F solubility limit achievable via conventional solid-state synthesis, namely, Li_1.25_Mn_0.2_Ti_0.55_O_1.85_F_0.15_ (LMTF15), and found that almost all of the F integrates into the DRX framework, with negligible F loss during the synthesis. Those encouraging results indicate that compositional tuning is an effective method to improve the fluorination and phase purity of DRX cathodes. We also devised a rapid, water-based washing procedure to reduce the impurity content in as-synthesized DRX samples and demonstrated the effectiveness of the method with LMTF25. Notably, the initial capacity and 25 cycle capacity retention of the cathode are improved by ca. 10 and 11%, respectively, following the water wash. Finally, we demonstrated the broader applicability of our analytical methodology by analyzing Li_1.33_Mn_0.33_Ti_0.33_O_1.33_F_0.66_ (LMTF66) prepared via mechanochemical synthesis. We find that the F solubility limit can be greatly enhanced with high-energy planetary milling of the precursor powders, with up to ca. 30% F incorporation (i.e., F_0.6_) into the DRX structure, compared to only <10% (i.e., F_<0.2_) achieved by conventional high-temperature sintering.

## Experimental Section

### Synthesis

Li_1.25_Mn_0.25_Ti_0.50_O_1.75_F_0.25_ (LMTF25) and Li_1.25_Mn_0.20_Ti_0.55_O_1.85_F_0.15_ (LMTF15) were prepared via solid-state synthesis. LMTF25 was obtained following a previously reported synthesis method.^26^ Both LMTF25 and LMTF15 were synthesized from a mixture of LiF (99.9%, Sigma-Aldrich), Li_2_CO_3_ (10 mol % excess, 99.9%, Sigma-Aldrich), MnO (99.99%, Sigma-Aldrich), and TiO_2_ (99% Anatase, Sigma-Aldrich). The precursor powders were first ball-milled together for 6 h at 300 rpm using a Retsch PM200 planetary ball mill. The mixed powder was then pelletized and fired in a furnace at 800 °C for 12 h with a ramp rate of 5 °C/min and cooled naturally to room temperature under a constant argon flow. As-synthesized pellets were then quickly transferred to an Ar-filled glovebox to limit air exposure. Li_1.33_Mn_0.33_Ti_0.33_O_1.33_F_0.66_ (LMTF66) was prepared by high-energy ball-milling following a previously reported procedure.^10^ In an Ar-filled glovebox, 1 g of a mixture of LiF (99.9%, Sigma-Aldrich), Li_2_O (10 mol % excess, 99.9%, Sigma-Aldrich), MnO (99.99%, Sigma-Aldrich), and TiO_2_ (99% Anatase, Sigma-Aldrich) was introduced into a 50 mL stainless steel jar along with five 10 mm and ten 5 mm stainless steel balls (Retsch, 0.5 and 4 g each, respectively). The jar was sealed under Ar, transferred to a planetary ball mill (Retsch PM200), and synthesis proceeded at 450 rpm for 40 h (30 min run with 5 min breaks, spinning direction reversed each time). After synthesis, the sealed jar was transferred to an Ar-filled glovebox and the powder sample was recovered.^27^

### X-ray Diffraction

Synchrotron X-ray diffraction (SXRD) patterns were collected at 11-BM at the Advanced Photon Source (APS) at Argonne National Laboratory, using the mail-in program. Room-temperature data were collected at λ = 0.458945 Å from 10 to 30° (2θ). Samples were placed in a 0.7 mm ID borosilicate glass capillary in an Ar-filled glovebox and then sealed using epoxy and modeling clay to avoid air exposure during shipping and analysis. Laboratory XRD patterns were collected on LMTF15, LMTF25, LMTF25w, and LMTF66 using a Panalytical Empyrean diffractometer with Cu Kα radiation (λ = 1.54178 Å) in reflection geometry. Samples were removed from an Ar-filled glovebox and placed on a zero-background silicon tray for measurement. LMTF66 was placed in an air-free holder before removal from the glovebox to prevent surface contamination. Diffraction data was collected from 10 to 80° (2θ). All resulting patterns were refined using the Rietveld method in TOPAS Academic v7.^27^

### Inductively Coupled Plasma Optical Emission Spectroscopy (ICP-OES) and Fluoride Ion-Selective Electrode (F-ISE) Analysis

Bulk chemical compositions were determined via ICP (Agilent 5800 ICP-OES) and using an F-ISE (Cole-Parmer). DRX powder samples were digested in a mixture of 65% nitric acid (Sigma-Aldrich, analytical grade) and 37% hydrochloric acid (Sigma-Aldrich, analytical grade) in a 4:1 (v/v) ratio and then diluted with ∼12 mL of distilled water for ICP measurement. For F-ISE measurements, the solutions were diluted by using a sodium acetate buffer and a fluoride ionic strength adjuster solution (TISAB, Cole-Parmer). The detailed protocol used for F-ISE measurements is presented in Note S1.

### Carbonate Titration

Carbonate titrations were performed using a custom-built titration mass spectrometry (TiMS) instrument that is nearly identical to that of a differential electrochemical mass spectrometry (DEMS) instrument, which has been described in previous publications.^28−30^ DRX powder samples (∼20 mg) were loaded in a custom-built, hermetically sealed titration vessel inside an Ar-filled glovebox. This vessel was then appropriately connected to the TiMS apparatus to avoid air exposure. During the experiment, the cell headspace was purged with 2 mL of Ar by the TiMS instrument every 4 min, and any accumulated gases were swept to the mass spectrometer chamber for analysis. The apparatus was calibrated for CO_2_ in Ar, allowing for the determination of the partial pressure of CO_2_ in each gaseous sample. After baseline levels corresponding to zero evolved CO_2_, 2 mL of a N_2_-sparged 10 M H_2_SO_4_ solution was injected into the titration vessel through a septum-sealed injection port. The resulting mixture was mixed with a magnetic stir bar. Gas samples were taken until the reaction was completed, as determined by the return of the CO_2_ signal to its baseline level. The amount of CO_2_ evolved was then quantified by using the known volume, temperature, and partial pressure of CO_2_ of each gas sample through the ideal gas law. Finally, the carbonate composition was calculated for each sample by using the sample mass, the amount of CO_2_ evolved, and the carbonate decomposition stoichiometry.

### Solid-State Nuclear Magnetic Resonance (ss-NMR) Spectroscopy

^7^Li and ^19^F ss-NMR spectra were recorded on LMTF25, LMTF25w, LMTF15, and LMTF66 at *B*_0_ = 2.35 T (100 MHz for ^1^H) using a Bruker BioSpin wide bore spectrometer equipped with a DMX 500 MHz console and a custom-made 1.3 mm X-broadband magic angle spinning (MAS) probe tuned to ^7^Li (38.9 MHz) or ^19^F (94.1 MHz). The ^7^Li and ^19^F ss-NMR spectra were obtained at 60 kHz MAS using a rotor-synchronized spin echo sequence (90°−τ_R_–180°−τ_R_) and 90° radio frequency (RF) pulses of 0.45 and 0.30 μs, respectively. To obtain a high-sensitivity ^7^Li and ^19^F ss-NMR data, a short (50 ms) recycle delay was used, and the data was averaged over 7168 and 15 360 transients, respectively. To obtain quantitative ^7^Li and ^19^F ss-NMR data, longer recycle delays of 20 or 5 s, respectively, were used, and the data was averaged over 32 or 240 transients. To avoid air exposure, all samples were packed in zirconia rotors in an Ar-filled glovebox and spun using dry nitrogen during data acquisition. Chemical shifts were externally referenced to pure lithium fluoride powder (LiF, δ_iso_(^19^F) = −204 ppm and δ_iso_(^7^Li) = −1 ppm).^20^ All ss-NMR spectra were processed using Bruker TopSpin 3.6.0 software. Spectral fits were carried out using an in-house python code and the Dmfit software.^31^ Additional background on solid-state NMR of DRX cathodes and details of the data fitting procedure can be found in Notes S2 and S3.

### Electrode/Cell Fabrication and Electrochemical Testing

Swagelok cells were assembled with a Whatman glass-fiber separator and 200 μL of commercial grade 1 M LiPF_6_ in ethylene carbonate (EC)/dimethyl carbonate (DMC) (50:50 v/v, Sigma-Aldrich) electrolyte solution. The as-synthesized cathode powder sample was mixed with carbon and a poly(tetrafluoroethylene) (PTFE) binder (Sigma-Aldrich) in a 70:20:10 mass ratio. To carbon coat the material, around 390 mg of active material and 110 mg of super C65 (Sigma-Aldrich) were ball-milled with five 10 mm stainless steel balls at 300 rpm for 6 h using a planetary ball mill. The carbon-coated material was then transferred to an Ar-filled glovebox and hand-ground with PTFE using a mortar and pestle for approximately 10 min. Hand-rolled films of cathode material were prepared with a loading density of about 6 mg active material/cm^2^ (around 2 mg of DRX active material per cell). Pure lithium metal (Sigma-Aldrich) was used as the counter electrode. All cells underwent galvanostatic testing using an Arbin BT2000 cycler between 1.8 and 4.7 V vs Li/Li^+^ at a rate of C/20.

### Washing Procedure for As-Synthesized DRX Powders

Details of the washing procedure and custom glassware used can be found in Note S4. Briefly, 300 mg of DRX powder sample was loaded into a custom glassware in an Ar-filled glovebox. Outside the glovebox, the loaded glassware was connected to a Schlenk line, and the DRX powder was continuously flushed through with N_2_ to avoid air and moisture exposure. The DRX powder sample was washed with 5 mL of outgassed DI (ODI) water. The N_2_ flow was kept on for 24 h after washing to dry the sample. The washed DRX sample was then further dried in the antechamber of an Ar-filled glovebox under dynamic vacuum overnight. The washed powder was recovered inside the glovebox with an ≈80% yield.

## Proposed Characterization Method

**Figure 1:**
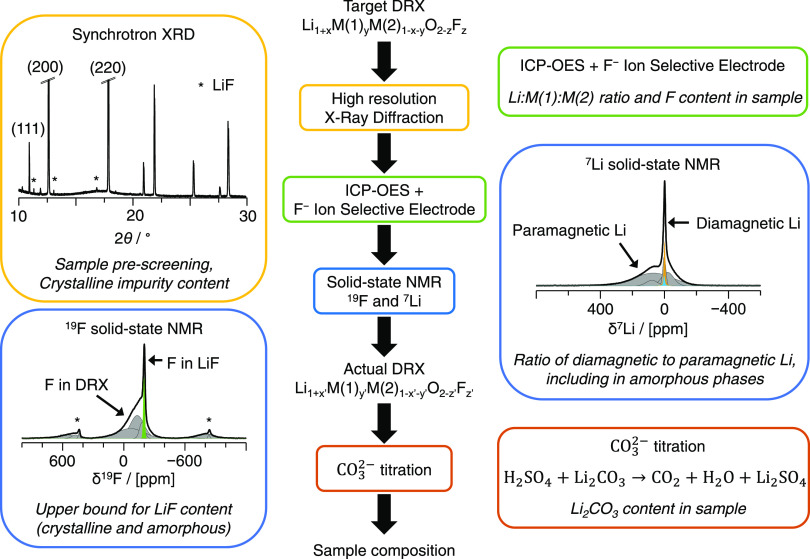
Proposed experimental methodology to assess the incorporation of F into the DRX phase and the composition of the sample.

The proposed experimental procedure to assess F incorporation into DRX cathodes and the composition of the as-synthesized powder sample is illustrated in Figure 1.

In the first step, XRD is conducted on the as-prepared sample. While laboratory powder XRD is a good choice for rapid screening of sample purity, its ability to identify minor and/or poorly crystalline phases is limited. Synchrotron XRD (SRXD) is generally preferred, as it provides higher sensitivity and resolution. Given the high calcination temperatures needed to form the DRX phase, any (lithium) transition-metal (TM) oxide or oxyfluoride phase (e.g., (Li−)TM–O, (Li−)TM–O–F) present in the sample is expected to be sufficiently crystalline to be identified and quantified via diffraction techniques and Rietveld refinements, even if present in small quantities. In contrast, some unreacted precursors, synthesis intermediates, and surface phases formed during sample handling in the glovebox (e.g., Li_2_CO_3_, LiF, Li_2_O, and LiOH) may be (partially) amorphous and thus only be observed via local structure probes. The subsequent discussion focuses on cases where no (Li−)TM–O or (Li−)TM–O–F phase other than the DRX phase of interest is present in the sample, which is achieved here by tuning the synthesis conditions.

In the second step, ICP-OES and F-ISE measurements are carried out and provide insight into the elemental ratio of Li, Mn, and Ti, and the total F content in the sample. We have not attempted to quantify the O content of the sample due to the presence of extraneous O in most ICP setups.

Next, ^7^Li and ^19^F ss-NMR are employed to distinguish and quantify Li and F species present in paramagnetic (DRX) and diamagnetic phases or domains in the sample. Species near paramagnetic centers (here, redox-active Mn), and therefore in the DRX phase, give rise to very broad and highly shifted ss-NMR resonances due to the strong paramagnetic interactions between the unpaired d electron spins on Mn and the ^7^Li or ^19^F nuclei under consideration (gray deconvolved signals in the spectra shown in Figure 1). In contrast, species present in diamagnetic impurity phases, or in Mn-poor regions of the DRX, do not suffer from paramagnetic broadening and give rise to significantly sharper resonances at discrete chemical shift values (yellow and green deconvolved signals in the ss-NMR spectra in Figure 1). More information regarding the ss-NMR properties of DRX cathode samples, and the determination of the Li and F molar fractions in DRX vs. impurity phases from the integrated intensities of the broad and sharp spectral components, can be found in Note S2. The Li and F contents in the entire sample obtained from ICP and F-ISE are then scaled by the Li/F molar fractions obtained from ss-NMR to obtain the absolute Li and F contents in the DRX phase. This method, while quantitative for Li, underestimates the amount of F in the DRX phase. Indeed, ^19^F species directly bonded to redox-active Mn species (more generally, to a paramagnetic TM) are NMR-silent as their corresponding ^19^F ss-NMR signals are too short-lived (and too broad as demonstrated for Li_1.15_Ni_0.45_Ti_0.3_Mo_0.1_O_1.85_F_0.15_) to be measured.^23^ As these NMR-silent F species are present only in the DRX phase, our method overestimates the amount of F-containing diamagnetic impurities, namely, LiF. Hence, ^19^F ss-NMR provides a lower bound for the amount of F in the DRX phase. An upper bound for the F content in the DRX phase can be obtained by considering the probability of forming F environments with no nearest-neighbor paramagnetic TM species, i.e., that can be observed by NMR, and scaling up the paramagnetic ^19^F signal intensity accordingly (more details in Note S2).

The method described above allows for the determination of the Li and F contents in the DRX phase. The overall stoichiometry of the cathode can then be determined if we assume no cation and no anion vacancies in the rock salt structure (those assumptions are further justified in Note S5). In an effort to optimize the synthesis of DRX cathodes, we also determined the nature and amount of all impurity phases in the sample. For this, carbonate titration using titration mass spectrometry (TiMS), a method developed by some of us,^28−30^ is used to obtain the amount of lithium carbonate in the sample (further justification as to why Li_2_CO_3_ is the only carbonate impurity considered in the as-synthesized samples is provided in Note S5). The total amount of Li in diamagnetic impurities is determined by scaling the Li content in the entire sample (obtained from ICP) by the fraction of the diamagnetic ^7^Li signal in the ss-NMR spectrum (see Note S2). This information is then combined with the LiF content in the sample obtained from ^19^F ss-NMR, and the Li_2_CO_3_ content derived from carbonate titration, to obtain the molar fraction of Li present in all of the phases in the sample, including DRX, LiF, Li_2_CO_3_, Li_2_O, and possibly LiOH formed from the reaction of Li_2_O with atmospheric moisture.

### Compositional Analysis of Li–Mn^2+^–Ti^4+^–O–F Cathodes Prepared by Solid-State Synthesis

#### Case Study of Li_1.25_Mn_0.25_Ti_0.50_O_1.75_F_0.25_ (LMTF25)

**Figure 2:**
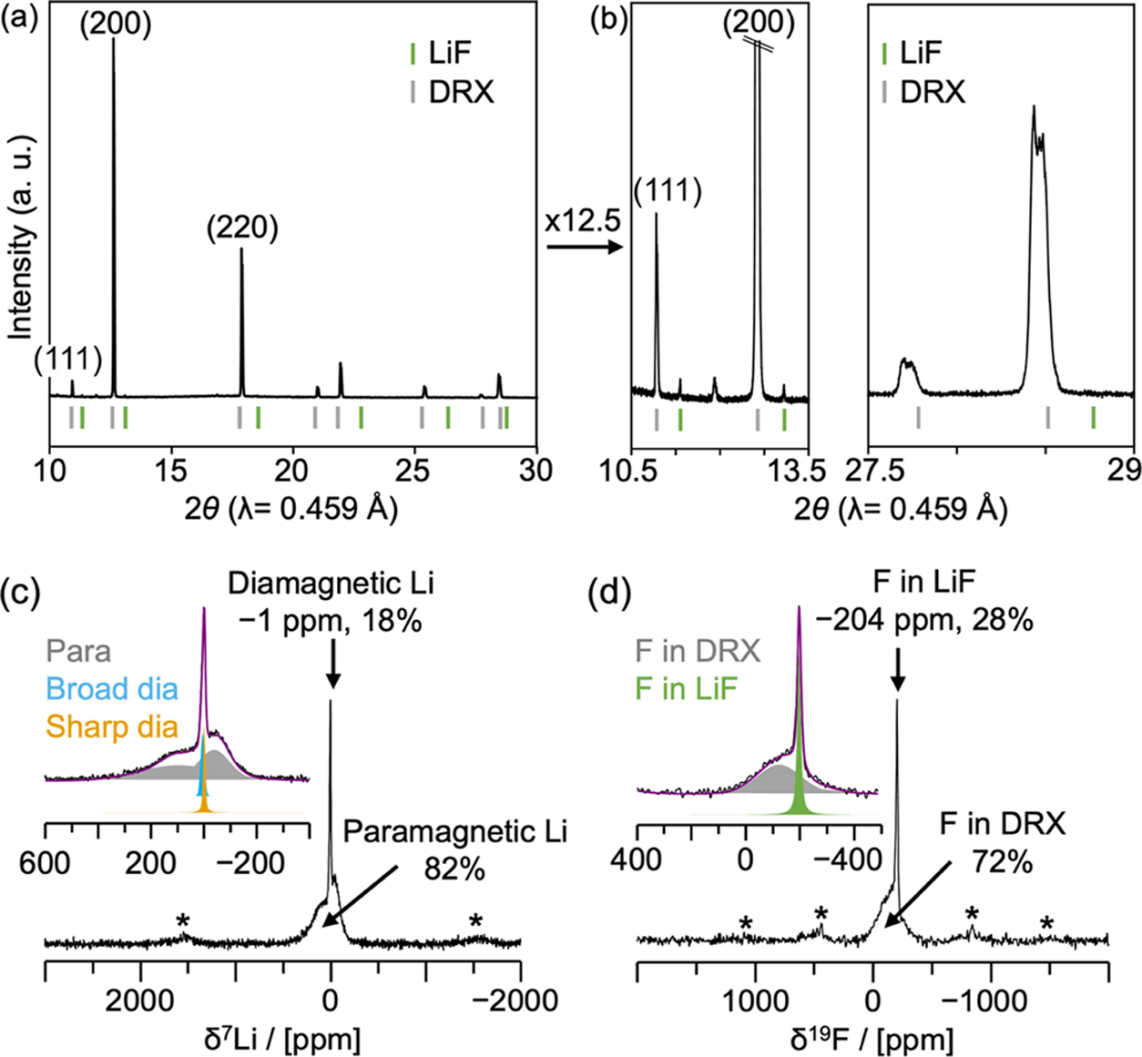
Structural characterization of the as-prepared Li_1.25_Mn_0.25_Ti_0.50_O_1.75_F_0.25_ (LMTF25) powder sample. (a) Synchrotron XRD data collected on an as-prepared LMTF25. (b) 12.5× enlargements of the SXRD pattern in the 10.5–13.5° 2θ region (left) and 27.5–29° 2θ region (right), highlighting reflections corresponding to crystalline LiF impurities (indexed in green) and the splitting of DRX reflections (indexed in gray for the target DRX composition) that indicates the presence of two DRX phases. The most intense DRX reflections are indexed by their (*hkl*) values. (c) ^7^Li and (d) ^19^F solid-state NMR spectra recorded on as-prepared LMTF25 at 2.35 T with recycle delays of 20 and 5 s, respectively, to ensure complete signal relaxation between scans. Spinning sidebands are indicated with an asterisk (*). The fraction of Li in paramagnetic and diamagnetic environments in the sample, as indicated in panel (c), is obtained from integration of the paramagnetic (gray) and diamagnetic (broader signal in light blue, sharper signal in orange) signals in the spectral deconvolution shown in the inset (overall fit in purple). Similarly, the fraction of F in DRX and LiF phases, as indicated in panel (d), is obtained from integration of the corresponding signals (DRX in gray, LiF in green) in the spectral deconvolution shown in the inset (overall fit in purple).

We first apply our methodology to a DRX cathode with target stoichiometry Li_1.25_Mn_0.25_Ti_0.50_O_1.75_F_0.25_ (LMTF25) initially reported by He et al.^26^ This material was prepared using a standard solid-state synthesis route, and 10% Li excess (as Li_2_CO_3_) to compensate for Li volatility during the 12 h calcination step at 800 °C (see the Experimental Section). Laboratory XRD analysis (see Figure S3a) of the as-synthesized LMTF25 powder sample indicates a single polycrystalline DRX phase with a cubic space group *Fm*3̅*m* and a lattice parameter *a* = 4.197 Å, consistent with laboratory XRD data reported previously.^26^ However, higher-resolution SXRD data on the same sample reveal a splitting of the DRX reflections, indicating the presence of two rock salt phases with slightly different lattice parameters (Figure 2a,b) and compositions. The unit cell parameters *a* for the two DRX phases, obtained from a Rietveld refinement of the SXRD pattern, are 4.171 and 4.178 Å, respectively, and those phases are present in an 89:11 ratio. The calculated Rietveld patterns are shown in Figure S4, and the full set of refined parameters obtained from the Rietveld analysis is given in Table S3. Using XRD alone, one could reach the conclusion that the two DRX phases account for almost all of the sample (99.5 wt %, equivalent to ∼98.8% of the Li), with crystalline LiF accounting for only 0.5 wt % (or 1.2% of the Li). As discussed below, further analysis using the methodology outlined in Figure 1 shows that the DRX phases instead account for only 90% of the Li in the sample. Given that our LMTF25 sample is slightly inhomogeneous in composition, we use a linear regression analysis (based on Vegard’s law) to estimate the degree of compositional fluctuation between the two DRX phases. We find the Mn content to vary between Mn_0.24_ and Mn_0.275_ around the target content of Mn_0.25_ in the two DRX phases, which should not significantly affect ss-NMR data quantification. We therefore adopt a pragmatic approach and seek to provide an average DRX stoichiometry for this sample.

Next, the LMTF25 sample was analyzed using ICP and F-ISE, with the results shown in Table S4. Since no Mn loss is expected at the calcination temperatures used in this work, cation stoichiometries obtained from ICP are referenced to the targeted Mn content, resulting in a Li/Mn/Ti ratio of 1.31:0.25:0.50. Interestingly, the final Li content in the sample (Li_1.31_) is greater than the targeted value (Li_1.25_), indicating that a 10 mol % Li excess overcompensates for Li volatility during the calcination step. ICP analysis indicates that ≈0.07 Li (≈5% of the total Li content) is lost during the synthesis, and the F-ISE measurement indicates that a similar molar amount of F is lost during the synthesis (≈0.08 F or ≈33% of the total F content). Hence, only 0.17 F per formula unit is present in the final sample instead of the targeted 0.25 F. The loss of equal amounts of Li and F during the synthesis (within measurement error) suggests LiF volatility at temperatures as low as 800 °C, which contrasts with Szymanski et al.’s findings that LiF loss proceeds directly after it melts at 848 °C in air (no flow).^32^ The low LiF volatilization temperature observed here may be explained by the use of a constant argon flow during the synthesis, which lowers the LiF partial pressure and its phase transition temperatures.

The Li and F molar fractions in the DRX phase were then derived from an analysis of the ^7^Li and ^19^F ss-NMR spectra shown in Figure 2c,d and fitted following a procedure described in Note S3. In each case, the overall fit is shown in purple, and an enlarged version of the isotropic region is shown as an inset with individual components obtained from the deconvolution represented in various colors as indicated. The fractions of Li in paramagnetic and diamagnetic environments, and of F in the DRX phase and in LiF impurities, are recorded in Table S5. 82% of the ^7^Li ss-NMR signal intensity is associated with paramagnetic environments, and therefore with the DRX phase, while the remaining 18% is attributed to diamagnetic environments, which could either arise from LiF, Li_2_CO_3_, Li_2_O, and/or LiOH impurities, or from Mn-poor regions of the DRX cathode. In fact, the relatively low Mn content in LMTF25 means that 9% of the Li in the DRX structure is expected to experience a diamagnetic environment, assuming a random cation distribution (see Note S2 and Table S2 for more details). Using this probability and the paramagnetic to diamagnetic ratio provided by the ^7^Li ss-NMR results, the total fraction of Li in the DRX structure is estimated to be 90% (with an estimated ±4% error from the fits derived from results from various fitting schemes), and the rest forms LiF, Li_2_CO_3_, Li_2_O, and/or LiOH impurities (see Table S6). ^19^F ss-NMR results reveal that, while most of the F is incorporated into the DRX structure (broad and overlapping resonances), 28% of the F forms LiF impurities in the sample, resulting in the sharp resonance centered around −204 ppm (see Note S2 and Table S2 for more details on the assignment). While this fraction is an upper bound for the amount of F present as LiF in the sample, since only a fraction of the F species in the DRX phase can be observed by NMR, a lower bound of 14% of F in LiF is obtained from the probability of forming NMR-visible F environments when cations are randomly distributed in the DRX structure (Note S2 and Table S7).

The combined ICP, F-ISE, and ss-NMR results allow us to compute the stoichiometry of LMTF25. Overall, we find that the Li and F contents in the DRX phase are below their target values of 1.25 and 0.25, respectively. The Li/Mn/Ti ratio in the DRX phase comes out as 1.22:0.26:0.52 after normalizing to the overall cation content of 2, while the F content lies within the range F_*x*_ (*x* = 0.12–0.15), with lower and upper bounds determined from an analysis of the ^19^F ss-NMR results (Table S5) and considering the probability of forming NMR-visible F environments in the DRX structure (Table S7). F incorporation into the bulk LMTF25 structure is clearly limited, and the final DRX stoichiometry comes out as Li_1.22_Mn_0.26_Ti_0.52_O_2–*x*_F_*x*_ with *x* = 0.12–0.15.

The overall sample composition was then deduced from a combined analysis of the ss-NMR, ICP, and carbonate titration results. The latter technique indicates a very small amount of Li_2_CO_3_ in the sample, on the order of 0.6 (±0.5) mmol Li_2_CO_3_/mol DRX (see Table S8), such that the fraction of the diamagnetic ^7^Li ss-NMR signal intensity associated with this phase can be neglected. The final distribution of Li (in Li mol %) among the DRX, LiF, Li_2_CO_3_, and Li_2_O/LiOH phases comes out as 90, 2–4, 0, and 6–8%, respectively, where the ranges provided here reflect the upper and lower LiF contents obtained from the analysis of the ^19^F ss-NMR results.

#### Case Study of Li_1.25_Mn_0.20_Ti_0.55_O_1.85_F_0.15_ (LMTF15)

Building upon our findings for LMTF25 and a F solubility limit in the range of F_*x*_ (*x* = 0.12–0.15) achievable through solid-state synthesis when the Li content is Li_∼1.25_, we set our next target DRX composition to Li_1.25_Mn_0.20_Ti_0.55_O_1.85_F_0.15_ (LMTF15), expecting to be able to synthesize this phase with a much higher phase purity. The results presented below were obtained on the pristine LMTF15 powder obtained under the exact same synthesis conditions as those for LMTF25.

Similarly to LMTF25, LMTF15 is composed of two rock salt phases, as indicated by laboratory and synchrotron XRD (Figure S3b,f, respectively). Yet, unlike LMTF25, the LMTF15 sample does not contain any crystalline impurity. Rietveld refinement of the SXRD pattern collected on the pristine powder sample yields unit cell parameters *a* = 4.154 and 4.164 Å for the two DRX phases present in a 52:48 ratio (see Table S3). Those unit cell parameters suggest a slight contraction of the DRX lattice as compared to LMTF25 (with lattice parameters *a* = 4.171 and 4.178 Å for the two phases), consistent with the lower Mn^2+^ (*r* = 0.83 Å) and higher Ti^4+^ (*r* = 0.605 Å) content in LMTF15. Once again, using a simple linear regression analysis based on Vegard’s law, we estimate the Mn content to vary by up to ±0.02 around the Mn_0.2_ target content in the two DRX phases. This compositional fluctuation is sufficiently small to justify the consideration of a single average DRX phase to simplify the analysis. Also, similarly to LMTF25, the 10 mol % Li excess used in the synthesis of LMTF15 is too high, resulting in a Li content of 1.30 per formula unit, as determined from ICP (see Table S4), instead of the targeted 1.25, but the Mn/Ti ratio (0.20:0.53) is very close to target. In contrast to LMTF25 where 33% F loss was recorded during synthesis, F-ISE results indicate a F stoichiometry of F_0.13_ for the LMTF15 sample (Table S4), corresponding to only 13% F loss during synthesis. F incorporation into the DRX structure is also much higher for LMTF15, with an upper bound for the amount of F present as LiF in the sample of 5% from ^19^F ss-NMR (Figure S5a and Table S5). A lower bound of 3% is obtained from the probability of forming NMR-visible F environments when cations are randomly distributed in the DRX structure (Table S7). Those results suggest that, when most of the F can be incorporated into the DRX structure, Li loss during synthesis no longer arises from LiF volatility but rather from Li_2_CO_3_ or Li_2_O volatility. ^7^Li ss-NMR indicates a large fraction of Li species in diamagnetic environments (22% of the total Li_1.30_ in the sample; Figure S5b), but since about 15% of the Li in the DRX structure (out of the Li_1.25_ content) is expected to experience a diamagnetic environment (Table S2), the total fraction of Li in the DRX structure is very high at 92% (with an estimated ±4% error from the fits), and the remaining 8% of the Li forms LiF (presumably amorphous since not observed by synchrotron XRD), Li_2_CO_3_, Li_2_O, and/or LiOH impurities (Table S6).

The combined ICP, F-ISE, and ss-NMR results allow us to compute the stoichiometry of LMTF15. The Li/Mn/Ti ratio in the DRX phase comes out as 1.24:0.21:0.55 after normalization, while the F content lies within a narrow range (F_*x*_ with *x* = 0.12–0.13; see Tables S5 and S7) and is close to target. The final DRX stoichiometry comes out as Li_1.24_Mn_0.21_Ti_0.55_O_2–*x*_F_*x*_ with *x* = 0.12–0.13. When it comes to determining the overall composition of the sample, carbonate titration indicates a very small amount of Li_2_CO_3_ impurity (5.6 ± 0.5 mmol of Li_2_CO_3_/mol of DRX; see Table S8), and the fraction of the diamagnetic ^7^Li ss-NMR signal intensity associated with this phase can once again be neglected. The final distribution of Li (in Li mol %) among the DRX, LiF, Li_2_CO_3_, and Li_2_O/LiOH phases comes out as 92, <1, 0, and 7%, respectively. Compared to LMTF25, more Li integrates into the DRX phase, the LiF impurity is drastically reduced, and the fraction of Li_2_O/LiOH remains the same. Those results clearly indicate that the Li_1.24_Mn_0.21_Ti_0.55_O_2–*x*_F_*x*_ (*x* = 0.12–0.13) composition (or 6% F substitution) is near the fluorine solubility limit for Mn^2+^/Ti^4+^-based DRX with a targeted Li content of 1.25 and prepared via a solid-state route using standard precursors.

#### Can a Water Wash Enhance the Purity of DRX Samples?

**Figure 3:**
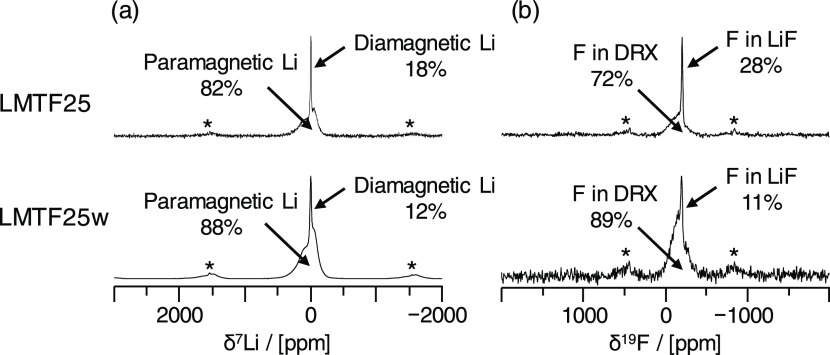
Comparison of ^7^Li and ^19^F ss-NMR spectra recorded on as-prepared LMTF25 (top) and water-washed LMTF25w (bottom). (a) ^7^Li and (b) ^19^F ss-NMR spectra were recorded at 2.35 T. Spinning sidebands are indicated with an asterisk (*). The percentage of paramagnetic and diamagnetic Li, and of F in DRX and LiF environments, was obtained from the integration of the corresponding deconvolved signals. The deconvolved ss-NMR spectra are shown in Figure 2c,d for LMTF25 and in Figure S6a,b for LMTF25w.

The two case studies presented so far reveal the presence of impurity phases in Li–Mn^2+^–Ti^4+^–O–F DRX cathode samples obtained by solid-state synthesis, mainly LiF and Li_2_O/LiOH, which have also been reported in previous work.^9,33,34^ Such impurities are poorly conductive and likely reduce cathode performance, but it is difficult to prevent their formation entirely by tuning synthesis parameters alone, as we have highlighted here, and we hypothesize that a postsynthesis powder washing procedure may further increase DRX phase purity. A simple washing procedure using deionized and degassed water has been reported for various NMC-type cathodes^35−37^ and is tested here on DRX compounds for the first time. The washing procedure is described in more detail in the Experimental Section and in Note S4. The effectiveness of the proposed washing process was assessed with LMTF25, as this sample exhibited significantly more impurities than the LMTF15 sample. Water-washed LMTF25 (LMTF25w) was analyzed by using our suite of characterization tools to determine the impact of the washing procedure on the DRX structure and stoichiometry and on the composition of the sample. While laboratory XRD shows a phase-pure material, SXRD indicates a small amount of crystalline LiF (Figure S3c,g, respectively). Rietveld refinement of the SXRD pattern indicates that the two DRX phases identified in the pristine sample are preserved upon washing, with only a slight expansion of their unit cell parameters from *a* = 4.171 and 4.178 Å before washing to *a* = 4.173 and 4.179 Å after washing; the ratio of the two phases changes more significantly from 89:11 to 79:21 upon washing. ICP and F-ISE analyses indicate a decrease in the Li and F contents in the sample upon washing, from Li_1.31_ and F_0.17_ to Li_1.28_ and F_0.12_, while the Mn and Ti contents are unaffected. ^7^Li and ^19^F ss-NMR analyses reveal similar spectral lineshapes for the LMTF25 and LMTF25w samples (relevant spectra overlaid in Figure 3), providing further evidence that the washing process does not significantly affect the bulk DRX structure.

A comparison of the deconvolved ^19^F ss-NMR spectra (Figures 2d and S6a for LMTF25 and LMTF25w, respectively) indicates a significant reduction of the fraction of F forming LiF impurities in the water-washed sample from 2 to 11% (noting again that those numbers are upper bounds for the fraction of F in LiF in the samples). A lower bound of 5% F in LiF impurities is obtained from the probability of forming NMR-visible F environments when cations are randomly distributed in the DRX structure (Table S7). Further, ^7^Li ss-NMR analysis reveals that the fraction of Li in diamagnetic environments is reduced from 18 to 12% of the total Li content in the sample upon washing (see Figures 2c and S6b and Table S5). Given that 9% of the Li in the DRX phase with composition LMTF25 is expected to be in a diamagnetic environment, the total fraction of Li in the DRX structure is estimated to be 97% (with an estimated ±4% error from the fits) and the remaining 3% of the Li forms LiF, Li_2_CO_3_, Li_2_O, and/or LiOH impurities (see Table S6). Taken together, ICP, F-ISE, and ss-NMR results allow us to derive a Li/Mn/Ti ratio of 1.25:0.25:0.50 after normalization, so exactly on target, and a F content of F_0.11_ (the uncertainty in the F content for this system is negligible, as shown in Tables S5 and S7). The average stoichiometry of the DRX phases in the washed sample comes out as Li_1.25_Mn_0.25_Ti_0.50_O_1.89_F_0.11_. Although the DRX Li content appears to have increased upon washing, this is very unlikely and the variation in the DRX Li content from pre- to postwash is within the estimated ±4% error from the fits of the ^7^Li ss-NMR spectra. Degassing of the deionized water used for washing the DRX sample is important as it prevents the accumulation of carbonate species at the surface of the sample, as testified by carbonate titration results indicating a fairly constant Li_2_CO_3_ impurity content (within measurement error), at about 0.3 (±0.5) mmol Li_2_CO_3_/mol DRX in the washed sample (see Table S8). The final distribution of Li (in Li mol %) among the DRX, LiF, Li_2_CO_3_, and Li_2_O/LiOH phases comes out as 97, 0–1, 0, and 2–3%, respectively, indicating a significant reduction in F- and Li-containing impurities in the sample.

**Figure 4:**
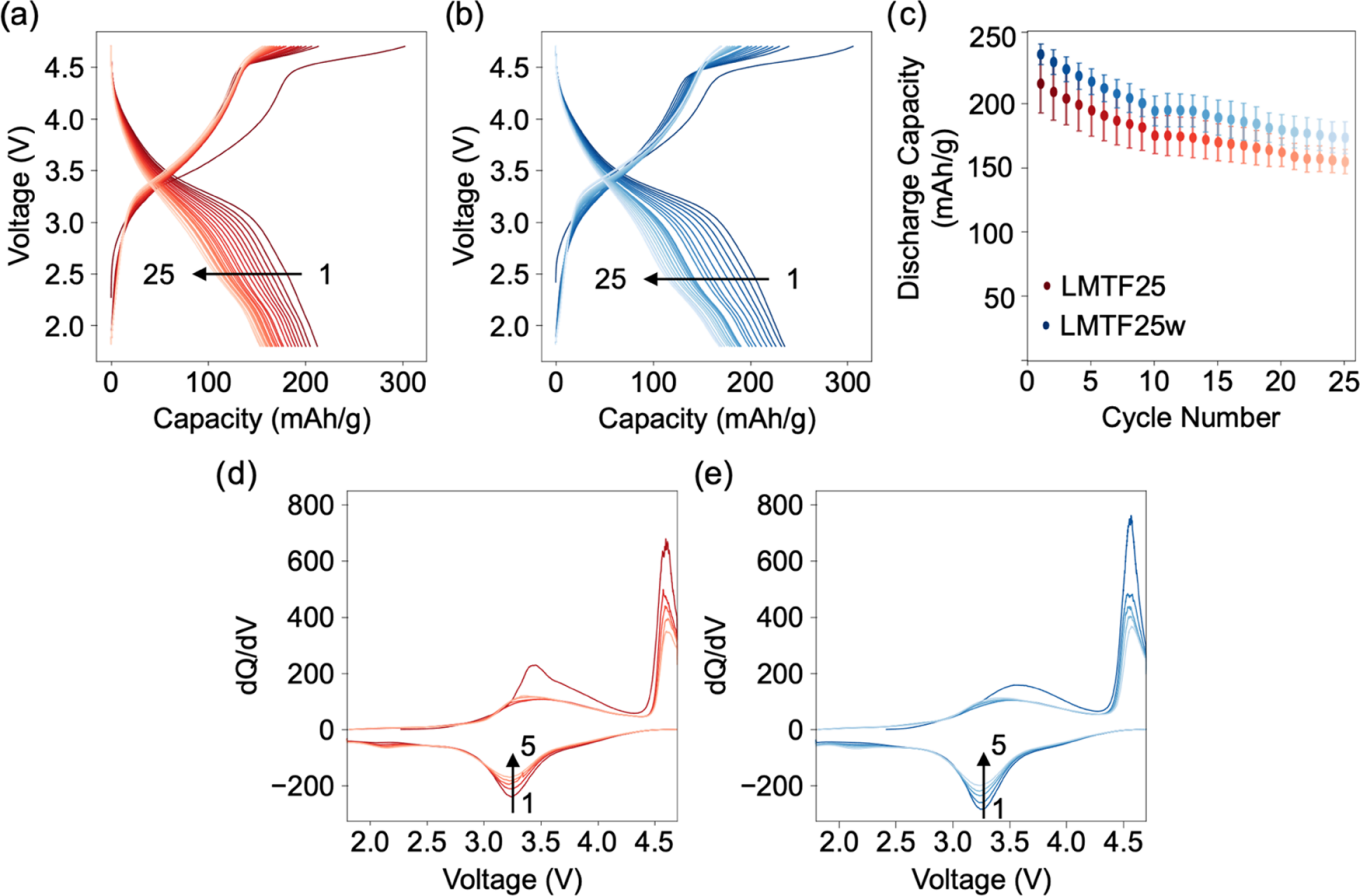
Electrochemical testing of as-prepared LMTF25 and water-washed LMTF25w in Li half-cells. Galvanostatic voltage profiles recorded for (a) LMTF25 and (b) LMTF25w over 25 charge–discharge cycles using a rate of 20 mA/g and a 1.8–4.7 V vs Li/Li^+^ voltage window. (c) Discharge capacity vs cycle number (averaged over three cells) and d*Q*/d*V* curves for (d) LMTF25 and (e) LMTF25w.

LMTF25 and LMTF25w cathodes were cycled in Li half-cells to assess the impact of the washing process on electrochemical performance. The cells were tested in galvanostatic mode (20 mA/g current density) over 25 charge–discharge cycles between 1.8 and 4.7 V vs Li/Li^+^, with results shown in Figure 4. The voltage profiles of representative LMTF25 and LMTF25w cathodes, shown in Figure 4a,b, are very similar and characteristic of DRX cathodes, with a smooth change in cell potential over most of the tested voltage window. A voltage plateau is observed above 4.5 V vs Li/Li^+^, with likely contributions from (i) electrolyte decomposition reactions at the surface of the oxidizing cathode (more pronounced during the first cycle) and (ii) anion redox processes, as has been suggested by several studies of DRX cathodes.^9,17,38^ Upon washing, this high voltage plateau lengthens, increasing both the charge capacity and reversible capacity on discharge, as shown in the plot of the discharge capacity vs cycle number in Figure 4c (averaged over three cells). The differential capacity (d*Q*/d*V*) plots shown in Figure 4d,e for the first five cycles of representative LMTF25 and LMTF25w cathodes confirm the overall very similar properties of the two cathode materials, with increased redox activity of the washed cathode near 4.5 V on charge and 3.25 V on discharge. We do not detect or expect any redox activity associated with LiOH or Li_2_O impurities upon cycling: LiOH degrades rapidly in an electrolyte environment (or might degrade during battery cycling in a fashion that would be difficult to detect in a voltage profile alone), and Li_2_O is not expected to be redox-active over the voltage window used here and will remain as surface species on the cathode.^39^ However, Li_2_O has a low Li-ion conductivity and its removal from the surface of the DRX particles is expected to reduce surface impedance, as well as dead weight from the cathode powder, both of which could explain the observed increased capacity after water washing.^40^ Overall, water washing results in a ca. 10% increase in the initial discharge capacity from LMTF25, from 210 to 232 mAh/g, and the discharge capacity of the water-washed LMTF25w cathode remains ca. 11% higher than that of its unwashed LMTF25 counterpart after 25 cycles. The average discharge voltage of the cell during the first cycle increases slightly upon washing, increasing from 3.12 V in the pristine state to 3.21 V in the washed state. The increase in reversible capacity and discharge voltage upon washing leads to a significant increase in energy density, from 664 Wh/kg for LMTF25 to 744 Wh/kg for LMTF25w. Further analysis of the redox processes and associated structural changes is needed to determine whether the changes in the electrochemical behavior observed upon washing result from the removal of Li-containing impurities (mostly LiF and Li_2_O/LiOH) from the surface of the DRX particles or from a change in the surface structure of the DRX particles, as has been observed for other surface treatment processes applied to DRX and NMC-type cathodes.^35,41^ Nevertheless, the present results are encouraging and indicate that washing DRX cathodes with water may be a viable process to improve electrochemical performance.

#### Compositional Analysis of a Li–Mn^2+^–Ti^4+^–O–F Cathode Prepared by Mechanochemical Synthesis

As mentioned earlier, increasing the amount of F in the DRX structure is advantageous, as it enables the incorporation of a greater fraction of low-valent redox-active TM species (e.g., Mn^2+^) on the cation lattice, which in turn increases the TM-based redox reservoir and reduces the need for poorly reversible and hysteretic anion-based redox. Various studies have demonstrated that highly fluorinated DRX cathodes can be prepared using high-energy ball-milling.^10,11,42^ In this section, we turn our attention to Li_1.33_Mn_0.33_Ti_0.33_O_1.33_F_0.66_ (LMTF66), a representative Li–Mn^2+^–Ti^4+^–O–F DRX composition with an impressive ∼260 mAh/g reversible capacity when cycled between 1.6 and 4.8 V vs Li/Li^+^, and with one of the highest F contents ever reported for a DRX.^10^ While previously reported ^19^F and ^7^Li ss-NMR results have indicated the presence of a significant amount of LiF and other Li-containing impurities in the as-synthesized sample, suggesting that the F content in the sample is well below target; quantitative insights into the composition of the DRX phase and sample are lacking, in part, due to the absence of further ICP and F-ISE analysis. By applying the methodology developed herein, we aim to identify the F solubility achievable via mechanochemical synthesis for Li–Mn^2+^–Ti^4+^–O–F compounds.

We replicated the high-energy ball-milling synthesis of LMTF66 reported by Lee et al.,^10^ which includes the use of 10% Li excess in the precursor mixture and planetary milling at 450 rpm for 40 h (details in the Experimental Section). Rietveld refinements of both laboratory XRD and SXRD patterns collected on the as-synthesized LMTF66 powder suggest a single rock salt phase with lattice parameter *a* = 4.208 Å (Figure S3d,h and Table S3) in excellent agreement with the previously reported value (*a* = 4.206 Å).^10^ Although neither pattern indicates the presence of crystalline impurities or of multiple DRX phases, the DRX reflections are significantly broadened as a result of the aggressive mechanochemical synthesis method, resulting in small particles (on the order of 100–200 nm) that are also poorly crystalline. Additionally, ball-milling may cause amorphization of the impurity phases commonly found in DRX samples, which undermines the utility of diffraction-based tools. Compositional analysis of LMTF66 using ICP reveals that 0.08 Li (≈5% Li) is lost during the synthesis, while F loss is minimal and within error from the F-ISE measurement (Table S4). The ^7^Li and ^19^F ss-NMR spectra shown in Figure S7 are composed of extremely broad and overlapping signals associated with the mechanochemically synthesized DRX phase. Although the ss-NMR resonances are broader than those in the previous cases because LMTF66 is both more disordered and more paramagnetic (contains a greater Mn content), the ^19^F ss-NMR results clearly indicate a significant proportion of LiF in the sample, as evidenced by the sharp diamagnetic resonance centered near −204 ppm (Figure S7a), corresponding to 12% of the integrated signal intensity. As this is an upper bound for the fraction of F in LiF, a lower bound of 4% is also obtained from the probability of forming NMR-visible F environments when cations are randomly distributed in the DRX structure (Table S7). Additionally, a sharp diamagnetic component can be resolved in the ^7^Li ss-NMR data (Figure S7b), corresponding to 21% of the integrated signal intensity, in excellent agreement with the diamagnetic ^7^Li signal intensity reported by Lee et al.^10^ Given that 3.8% of the Li in the DRX phases is expected to be in a diamagnetic environment (Table S2), the total fraction of Li in the DRX structure is estimated to be 82% (with an estimated ±4% error from the fits), and the remaining 18% of the Li forms LiF, Li_2_CO_3_, Li_2_O, and/or LiOH impurities (see Table S6). Taken together, ICP, F-ISE, and ss-NMR results allow us to derive a Li/Mn/Ti ratio of 1.27:0.37:0.36 after normalization, which is almost on target and a F content in the range of F_*x*_ with *x* = 0.56–0.62 (see Tables S5 and S7). The stoichiometry of the DRX phase comes out as Li_1.27_Mn_0.37_Ti_0.36_O_2–x_F_*x*_ with *x* = 0.56–0.62. The amount of F that can be incorporated into the bulk DRX lattice is drastically increased compared with the LMTF15 and LMTF25. This may be attributed to the high “equivalent temperature” reached during the ball-milling process from shear stresses,^24^ resulting in entropic stabilization of a more highly fluorinated DRX composition and to the use of a closed vessel synthesis method minimizing F volatility. Carbonate titration analysis reveals the presence of 4.4 (±0.5) mmol of Li_2_CO_3_/mol of DRX in the LMTF66 sample (see Table S8), which is negligible, and the final distribution of Li (in Li mol %) among the DRX, LiF, Li_2_CO_3_, and Li_2_O/LiOH phases comes out as 82, 2–6, 0, and 12–16%, respectively.

## Discussion

Given the large compositional space available for exploration, the rapid development of DRX cathodes hinges on the establishment of precise and robust material design rules. Much work has already been devoted to better understanding the impact of fluorination on the capacity retention,^14,43,44^ voltage hysteresis,^45^ and power capability of DRX cathodes,^17,46^ yet those studies have assumed that the target DRX composition is achieved during synthesis. The present work instead proposes a broadly applicable method to assess the degree to which F incorporates into the bulk DRX structure as well as the composition of the as-synthesized DRX powder sample. The results obtained on Li–Mn^2+^–Ti^4+^–O–F DRX compounds prepared via standard solid-state and mechanochemical milling synthesis reveal that complete fluorine incorporation into the DRX structure is rarely achieved. Rather, fluorination is limited to <10% (F_<0.2_) for solid-state synthesis, while high fluorination levels up to ∼30% (F_0.6_) can be achieved through high-energy ball-milling. The inability to achieve high fluorination via standard solid-state synthesis stems in part from a highly stable LiF precursor (or synthesis intermediate),^32,47^ locking in both Li and F during the synthesis, which is evidenced by the presence of LiF in all of the as-prepared Li–Mn–Ti–O–F cathodes of interest to this work. Moreover, LiF is volatile at the high temperatures (800–1000 °C) required for DRX synthesis, explaining the net loss of Li (≈5% loss for LMTF25 and LMTF15) and F (≈33% loss for LMTF25 and ≈13% loss for LMTF15), in good agreement with prior work.^32^ The combination of LiF stability and volatility explains why the targeted F content is difficult to reach by solid-state synthesis, leading to average Mn oxidation states >2, although the stoichiometry of the optimized LMTF15 compound is remarkably close to the targeted one (see Table 1). When it comes to mechanochemical synthesis, our analysis of the LMTF66 compound suggests a similar ≈5% Li loss during synthesis but remarkably no F loss, likely due to the use of a closed vessel synthesis method minimizing F volatility. In fact, the average Mn oxidation state for this compound is very close to 2 at the lowest possible fluorination level (F_0.56_, see Table 1), suggesting that the actual F content is close to this value. In all cases, a 10% excess Li in the precursor mixture to compensate for Li loss during the synthesis is too high, resulting in Li-containing impurities in the final sample, with a particularly significant amount of Li_2_O/LiOH impurities in mechanochemically synthesized LMTF66 (accounting for 12–16% of the Li in the sample).

**Table 1:** Calculated DRX Stoichiometry Results from Combined ICP, F-ISE, ^7^Li, and ^19^F ss-NMR Results and Mn Oxidation State Range Assuming a Cation and Anion Stoichiometry of 2 and a Ti Oxidation State of 4+

target composition	Li	Mn	Ti	min. F	max. F	Mn ox. st. range
Li_1.25_Mn_0.2_^2+^Ti_0.55_O_1.85_F_0.15_ (LMTF15)	1.24	0.21	0.55	0.12	0.13	2.06–2.07
Li_1.25_Mn_0.25_^2+^Ti_0.50_O_1.75_F_0.25_ (LMTF25)	1.22	0.26	0.52	0.12	0.15	2.16–2.25
Li_1.25_Mn_0.25_^2+^Ti_0.50_O_1.75_F_0.25_ (LMTF25w)	1.25	0.25	0.50	0.11	0.11	2.50–2.53
Li_1.33_Mn_0.33_^2+^Ti_0.33_O_1.33_F_0.66_ (LMTF66)	1.27	0.37	0.36	0.56	0.62	1.97–1.83

The present work also proposes a simple procedure to wash soluble impurity phases off the surface of DRX particles using outgassed deionized (ODI) water. This procedure effectively reduces the amount of Li-containing diamagnetic impurities from 10 to 3% of the total Li molar content in the sample (see Table S6) and drastically reduces the amount of LiF impurities (which account for 14–28% of the total F in LMTF25 and 5–11% of the total F in LMTF25w). Although it also slightly reduces the Li and F contents in the DRX phase and increases the average Mn oxidation state (see Table 1), a rapid water wash has a positive impact on the electrochemical performance as it increases both the initial reversible capacity (by ca. 10%) and the capacity retention (by ca. 11% after 25 cycles). Notably, the development of improved compositional analysis methods for DRX cathodes, and methods to reduce the impurity content in the samples, could resolve an ongoing debate on the impact of LiF impurities on the capacity and long-term cyclability of DRX cathodes. While LiF is insulating in nature, potentially hindering Li^+^ transport and charge transfer at the DRX/electrolyte interface,^48^ it has also been proposed as a protective barrier against electrolyte oxidation at the surface of high voltage cathodes^49^ and may reduce reactivity when DRX cathodes are charged to potentials ≥4.5 V vs Li/Li^+^.

The compositional analysis method proposed in this work is broadly applicable to DRX cathode samples and can provide a good estimate of the stoichiometry of most DRX compounds without requiring access to national research facilities (beamlines) nor advanced computational simulations. The method can be further simplified by omitting the carbonate titration step without losing any information about the stoichiometry of the DRX phase. We see two possible ways of improving the current method, with the caveat that these more accurate methods require either advanced computational simulations or beamline experiments and are therefore not practical for high-throughput synthesis optimization. First-principles cluster expansion Monte Carlo simulations can provide statistics on the distribution of Li and F environments in the DRX phase for a given composition and at a given synthesis temperature. These results would remove any ambiguity as to the assignment of the ss-NMR signals (e.g., related to the presence of diamagnetic Li sites or NMR-silent F species in the DRX phase) and allow for the exact F content in the DRX phase to be obtained, a clear improvement as compared to the ranges of F content presented in the present work. Additionally, hard X-ray absorption spectroscopy (XAS) can lead to a more precise quantification of the stoichiometry of the DRX phase by providing information on the average oxidation state of the TM species (here, Mn).

## Conclusions

We have devised a multistep experimental method to assess fluorine incorporation into the bulk DRX cathode structure and the composition of DRX samples prepared via solid-state and mechanochemical synthesis, combining X-ray diffraction (XRD), inductively coupled plasma (ICP) and fluoride ion-selective electrode (F-ISE) analyses, solid-state nuclear magnetic resonance (ss-NMR), and carbonate titration. This methodology was used to optimize the solid-state synthesis of Li–Mn^2+^–Ti^4+^–O–F cathodes, greatly enhancing F incorporation into the DRX structure. It also confirmed the increase in F solubility that can be achieved through mechanochemical synthesis, presumably due to the use of a closed synthesis vessel preventing F loss during synthesis and the high shear forces (or “equivalent temperatures”) that can be achieved with high-energy planetary milling. Overall, for Mn- and Ti-based DRX, we find that fluorination is limited to <10% (F_<0.2_) for solid-state synthesis, while high fluorination levels up to ∼30% (F_0.6_) can be achieved through high-energy ball-milling. In addition, this work proposes a water-based washing procedure to remove impurities such as LiF, Li_2_CO_3_, and Li_2_O/LiOH from as-synthesized DRX powder samples. This low-cost and scalable washing procedure was tested on the Li_1.25_Mn_0.25_Ti_0.50_O_1.75_F_0.25_ DRX cathode, resulting in a decrease in the amount of Li-containing diamagnetic impurities from 10 to 3% of the total Li molar content in the sample and a significant decrease in the LiF impurity content. The rapid water wash was also found to have a positive impact on the electrochemical performance as it increased both the initial reversible capacity (by ca. 10%) and the capacity retention (by ca. 11% after 25 cycles). Overall, this work highlights the need for more accurate reports of the stoichiometry of DRX materials to accelerate the deployment of this promising class of Li-ion cathodes, which depends on the inclusion of ICP, F-ISE, and solid-state NMR in the analytical framework.

## Supporting Information Available

Additional information on the characterization methods used in this study: protocol for fluoride ion-selective electrode measurements, background on solid-state NMR of DRX cathodes (challenges, quantification, and data analysis), details of the washing procedure for as-synthesized DRX powders, and an explanation of how the stoichiometry of the DRX phase and the overall sample composition are obtained; additional data: XRD (laboratory and synchrotron) analysis, elemental analysis, solid-state NMR analysis, and carbonate titration of Li–Mn–Ti–O–F samples (PDF)
