# Supplementary material for: An Experimental Approach to Assess Fluorine Incorporation into Disordered Rock Salt Oxide Cathodes
Source: Chem Mater. 2024 Apr 3;36(8):3643–54. doi: 10.1021/acs.chemmater.3c03138 (PMC11044264; doi:10.1021/acs.chemmater.3c03138)
Supplement: Supplementary file 1 — cm3c03138_si_001.pdf [file cm3c03138_si_001.pdf]

An experimental approach to assess fluorine incorporation into disordered rocksalt oxide cathodes

Supplementary Information

Raynald Giovine, [ǂ,a,b] Eric Yoshida, [ǂ,a,b] Vincent C. Wu, [ǂ,a,b] Yuefan Ji,[a,b] Matthew J. Crafton,[c] Bryan D. McCloskey,[c,d] and Raphaële J. Clément[*,a,b]

a. Materials Department, University of California Santa Barbara, California 93106, United States
b. Materials Research Laboratory, University of California Santa Barbara, California 93106, United States
c. Department of Chemical and Biomolecular Engineering, University of California Berkeley, CA 94720, USA
d. Energy Storage and Distributed Resources Division, Lawrence Berkeley National Laboratory, Berkeley, CA 94720, USA

Corresponding Author: R.J.C., rclement@ucsb.edu

Author contribution: [ǂ]R.G., E.Y., and V.C.W. contributed equally.

Supplementary Note 1: Protocol for fluoride-ion selective electrode measurements

Fluoride-ion selective (F-ISE) electrodes measure the amount of disassociated F^- ions in a solution. Proper preparation of solutions for F-ISE is required, as several factors may affect the quantification. As the concentration of non-fluoride species may impact the measurement, a total ionic strength adjustment buffer (TISAB) is used to minimize variation between samples and standards. Additionally, a pH buffer is required to maintain the pH within the operable range. Below pH 5.5, HF and HF_2^- species form, reducing the activity of fluoride-ions. At high pH, the high concentration of OH^- ions in solution may also interfere with the measurement.[1] Thus, we target a pH of 6.0 for our measurements.

A combined TISAB + pH buffer stock solution used to dilute all sample and reference solutions was first prepared. The TISAB solution consisting of a mixture of acetic acid, NaCl, cyclohexylenedinitrilotetraacetate (CDTA), and NaOH was purchased from Cole Parmer and used as received. A 15 wt.% solution of sodium acetate in H_2O used as pH buffer was prepared in-house. The combined buffer solution was obtained by mixing the TISAB and the 15% sodium acetate solutions in a 9:1 mass ratio. The concentration of the sodium acetate solution, and the ratio of the two solutions, were chosen so as to provide a pH close to 6.0 for the final, diluted solutions.

Reference samples were prepared by digesting LiF in a 4:1 solution of HNO_3 and HCl (by volume) to matrix match the dissolution of DRX samples. Distilled water was used to sequentially dilute the solution to generate 100 ppm, 10 ppm, and 1 ppm standards. The standards were then diluted using the combined buffer solution, with a buffer:standard solution ratio set to 15:1 by mass. A calibration curve was generated through F-ISE measurements on the three standard solutions, where a linear fit was obtained by plotting log(F,ppm) as a function of the inverse potential reading.

For the preparation of the DRX-containing solutions, ~10 mg of DRX powder sample was digested overnight in a solution of 4 mL HNO_3 and 1 mL HCl. ICP and F-ISE measurements were carried out on the same digested "mother solution". For F-ISE, around 0.5 g of the mother solution was first diluted using 2 mL of H_2O, after which the combined buffer solution was added in a 15:1 mass ratio. The F ppm content was determined from the F-ISE probe measurement and using the calibration curve.

Supplementary Note 2: Background on solid-state NMR of DRX cathodes

Challenges associated with solid-state NMR of DRX cathodes. ^{7}Li and ^{19}F solid-state NMR (ss-NMR) of DRX cathodes presents significant challenges for two reasons:

1) The paramagnetic nature of these materials, resulting from the high concentration of redox-active TM species (here, Mn ions) containing unpaired electrons in their *d* orbitals.[2] Such electrons interact strongly with nearby nuclear spins via through-space dipolar interactions that result in short ss-NMR signal lifetimes and significant broadening of the ss-NMR lines. Moreover, through-bond Fermi contact interactions resulting from the delocalization of unpaired electron spin density from the TM *d* orbitals to the *s* orbitals of the nucleus under study (here, ^{7}Li or ^{19}F), via bridging O *2p* orbitals in the case of ^{7}Li, leads to chemical shifts on the order of hundreds or even thousands

of ppm.[2,3] While through-space paramagnetic dipolar interactions are relatively long-range (they decay as $\frac{1}{r^3}$, where r is the distance from the paramagnetic center), the Fermi contact interaction is short range and depends on the bond connectivity of the nucleus of interest with paramagnetic centers up to ≈5 Å away.[4,5] In DRX cathodes, paramagnetic TMs in the first two cation coordination shells around Li, and in the first three cation coordination shells around F, will give rise to a non-zero Fermi contact shift, as shown in **Figure S1**. As mentioned in the main text, F directly bonded to a redox-active TM species are NMR-silent as their corresponding ^{19}F ss-NMR signals are too short-lived to be observed. ^{19}F nuclei in the DRX phase with a paramagnetic TM in the second and/or third coordination shell can be observed and quantified, but the presence of NMR-silent F species leads to an overall underestimation of the amount of F in the DRX phase from ^{19}F ss-NMR. In contrast, Li is at least two bonds away from the redox-active TM, resulting in weaker paramagnetic interactions and fully quantitative ^{7}Li ss-NMR spectra.

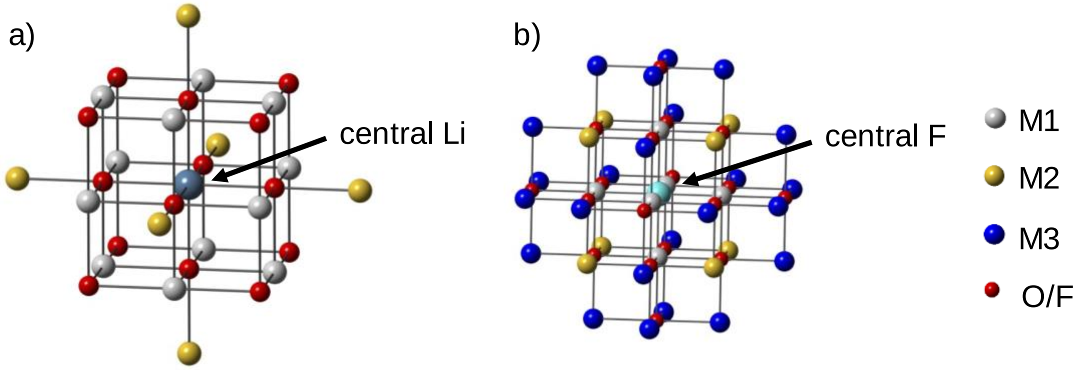

Figure S1. Local environments for (a) Li and (b) F in DRX cathodes. Paramagnetic transition metals (TMs) in the cation coordination shells depicted in grey (M1, 1st shell), yellow (M2, 2nd shell), and blue (M3, 3rd shell) are within 5 Å from the central nucleus and will contribute to its Fermi contact shift. The Fermi contact shift contribution from each open-shell TM depends on the geometry of the through-bond interaction pathway. For example for Li in a) this contribution depends on whether the TM-O-Li bond angle is approximately 180° or 90°, and on the TM-O and O-Li bond lengths.

2) The disordered nature of DRX compounds results in a large number of Li and F local environments, which in turn leads to a distribution of ^{7}Li and ^{19}F ss-NMR resonances with closely-spaced chemical shifts.

The compounded effects of paramagnetic broadening and of a wide chemical shift distribution leads to a large number of broad and overlapping resonances in the ^{7}Li and ^{19}F ss-NMR data. The resulting very broad ^{7}Li and ^{19}F ss-NMR spectra are typically asymmetric, indicating a skewed distribution of local environments consistent with the presence of short range order in these systems.[6–8] Unfortunately, individual resonances cannot be resolved and therefore attributed to specific local environments in the DRX structure, unlike layered and spinel oxide cathodes for which $^{6/7}$Li ss-NMR has proven extremely informative to identify local cation (dis)ordering and defects.

Identifying DRX ^{7}Li and ^{19}F environments in the sample. For the purpose of determining the phase makeup / composition of a DRX cathode sample, ^{7}Li and ^{19}F ss-NMR provide essential

information on the distribution of Li and F local environments in the sample. On the one hand, broad (e.g., with a full width at half maximum or FWHM ≥ 100 ppm for each component signal) and highly shifted $^{7}Li/^{19}F$ ss-NMR resonances correspond to Li/F local environments in the vicinity of paramagnetic Mn species, i.e., in the DRX phase, while sharp $^{7}Li/^{19}F$ ss-NMR resonances (e.g., FWHM ≤ 30 ppm) are associated with diamagnetic Li/F local environments. The latter can be defined as having *all* of the cation sites in the various coordination shells depicted in **Figure S1** be occupied by diamagnetic species (here, Li^{+} or Ti^{4+}). Diamagnetic $^{7}Li/^{19}F$ environments either correspond to: (a) Li/F-containing diamagnetic impurity phase (LiF, Li_2CO_3, Li_2O or LiOH), resulting in resonances near 0 ppm for ^{7}Li, and near −204 ppm for ^{19}F, as listed in **Table S1**, and (b) in the presence of Mn-poor domains within the DRX structure, which also leads to ^{7}Li and ^{19}F resonances near 0 ppm and −204 ppm, respectively.

Table S1. ^{7}Li and ^{19}F ss-NMR chemical shifts reported for pure LiF, Li_2CO_3, Li_2O and LiOH.

	δ_{iso} (^{7}Li) / ppm	δ_{iso} (^{19}F)/ ppm	reference
LiF	−1.4	−204.0	Ménétrier *et al.*[9]
Li_2CO_3	0.0	N/A	Meyer *et al.*[10]
Li_2O	2.8	N/A	Meyer *et al.*[10]
$LiOH.H_2O$	0.4	N/A	Meyer *et al.*[10]

Given the difficulty of separating the DRX and impurity phase contributions to the observed diamagnetic signals, we have computed the probability of forming diamagnetic ^{7}Li and ^{19}F environments within the DRX phase for the compositions of interest to this work, and assuming a random distribution of cations (results in **Table S2**). We note that, while most DRX cathodes exhibit some amount of short-range order due to the comparatively low formation energy of Li-F bonds as compared to redox-active TM-F bonds,[11,12] the formation of diamagnetic Li sites (as probed by ^{7}Li ss-NMR) depends on the longer-range structure (up to 4 bonds away from the central Li), and the random cation model used here provides a good estimate of the fraction of diamagnetic Li environments in the sample. When it comes to ^{19}F, the probability of forming diamagnetic local environments is very low for all DRX compounds (≤ 1.8%) considered here, and it is therefore safe to assume that all diamagnetic ^{19}F resonances arise from LiF impurities in the sample. On the other hand, the probability of forming diamagnetic ^{7}Li environments within the DRX phase is non-negligible for the LMTF15 (15%), LMTF25 (9%), and LMTF66 (3.8%) compositions, indicating that the diamagnetic ^{7}Li ss-NMR resonances in the corresponding spectra may be attributed to Li in diamagnetic impurity phases (LiF, Li_2CO_3, Li_2O or LiOH) and/or within Mn-poor DRX domains. The following equations were used to calculate the probabilities *P(X, Dia)* of forming a diamagnetic Li or F environment in a DRX structure assuming randomly distributed cation and anion sites:

$$P(Li, Dia) = (1 - x_{Mn})^{18}$$
$$P(F, Dia) = (1 - x_{Mn})^{38}$$

where x_{Mn} is the overall fraction of Mn in DRX cation sites. The exponential factors in the above equations correspond to the number of cation sites within a 5Å distance from a central Li or F site, which corresponds to 18 and 38, respectively (see Figure S1).

Table S2. Probability (P) of forming diamagnetic Li and F environments in the DRX phase, assuming a random distribution of cations in the rock salt structure and a target DRX composition.

Composition	P(Li, Dia) in %	P(F, Dia) in %
$Li_{1.25}Mn_{0.25}Ti_{0.50}O_{1.75}F_{0.25}$ (LMTF25)	9.0	<0.01
$Li_{1.25}Mn_{0.20}Ti_{0.55}O_{1.85}F_{0.15}$ (LMTF15)	15.0	0.02
$Li_{1.33}Mn_{0.33}Ti_{0.33}O_{1.33}F_{0.66}$ (LMTF66)	3.8	<<0.01

Quantification. ^{7}Li ss-NMR analysis of a DRX sample can provide quantitative information on the fraction of paramagnetic vs. diamagnetic Li-containing phases, but the ^{7}Li chemical shift range spanned by LiF, Li_2CO_3, Li_2O, and LiOH is smaller than the width of the diamagnetic resonance and those phases cannot be distinguished on the basis of ^{7}Li ss-NMR alone. ^{19}F ss-NMR, on the other hand, can unambiguously identify LiF with its characteristic resonance at −204 ppm, but only provides semi-quantitative information on the molar fraction of F in LiF and in the DRX phase. In all of our quantitative NMR analyses, care was taken to obtain fully relaxed ^{7}Li and ^{19}F ss-NMR spectra, by ensuring that the magnetization of all of the spins in the sample returned to their equilibrium orientation (along the z axis or the direction of the external magnetic field) between scans. The timescale of such a process follows the longitudinal (T_1) relaxation time. While typical T_1 values for ^{7}Li and ^{19}F nuclei in pure LiF, Li_2CO_3, Li_2O, or LiOH samples are on the order of 60 s at the low magnetic field used in this work (2.35 T), these T_1 values were found to be drastically reduced to < 1 s for ^{7}Li and < 5 s for ^{19}F in similar phases/environments present in the samples of interest to this work. These much shorter T_1 times result from paramagnetic relaxation enhancements caused by the nearby DRX phase containing open-shell Mn ions. The T_1 values of the diamagnetic ^{7}Li and ^{19}F environments in the various DRX samples were estimated from a series of spin-echo spectra collected upon systematically increasing the inter-scan (or recycle) delay and monitoring the change in the intensity of the diamagnetic signals. Here, all spectra used for quantification had reached a plateau in the diamagnetic signal intensity behavior, indicating that the inter-scan delay (called d_1 on a Bruker spectrometer) was sufficiently long for all signals to be fully relaxed ($d1 \geq 5*T_1$).

As explained earlier, ^{19}F ss-NMR provides a lower bound for the amount of F in the DRX phase. An upper bound can be obtained by considering the probability of forming F environments in the DRX phase with no nearest-neighbor paramagnetic TM species, $P(F, \mathrm{M1} \neq \mathrm{Mn}) = (1 - \frac{S_{Mn}}{2})^6$, where S_{Mn} is the Mn stoichiometry in the DRX phase, and $\frac{S_{Mn}}{2}$ is the probability of $\mathrm{M1} = \mathrm{Mn}$ for a single $\mathrm{M1} - \mathrm{F}$ bond when the overall cation stoichiometry is 2. Computing P(F, M1≠Mn) assuming a random distribution of cations in the rock salt structure provides a lower bound for the fraction of NMR-observable F in DRX structure, given that any short-range order present in the material will tend to minimize the number of high energy redox-active TM-Li bonds and increase P(F, M1≠Mn). The resulting adjusted intensity of the paramagnetic ^{19}F ss-NMR signal can be derived as $p' = \frac{p}{P(F, \mathrm{M1} \neq \mathrm{Mn})}$, where p is the integrated intensity of the observed paramagnetic (DRX) ^{19}F ss-NMR signal. The upper bound for the fraction of F in the DRX phase can then be computed as: $\frac{p'}{p'+d}$, where d is the integrated intensity of the diamagnetic ^{19}F ss-NMR signal. P(F,

M1≠Mn) values computed from the Mn stoichiometries derived from the multi-step compositional analysis of the DRX cathodes of interest to this work are provided in **Table S7**.

Supplementary Note 3: ^{7}Li and ^{19}F ss-NMR data analysis

^{7}Li and ^{19}F spin echo spectra reported in this study were deconvolved using a sum of pseudo-Voigt functions to recreate the overall line shape using an in-house python code and the DMfit software package[13]. Each pseudo-Voigt function is a linear combination of a Gaussian and a Lorentzian function controlled by the Gaussian/Lorentzian ratio, $0 \leq GL \leq 1$. The number of components (functions) used in our models was kept to a minimum.

^{7}Li or ^{19}F ss-NMR signals associated with local environments in the DRX structure were fitted using pure Gaussian functions (GL = 1) to model a distribution of chemical shifts. Diamagnetic ^{7}Li and ^{19}F ss-NMR signals were fitted using one or two pseudo-Voigt functions. In all cases, the position (chemical shift), intensity, and full-width at half maximum (FWHM) of the component functions were systematically varied during the fitting procedure. We note that the final chemical shift of the diamagnetic components remained within ± 5 ppm (^{7}Li) and ± 14 ppm (^{19}F) of the chemical shifts listed in **Table S1**, and their FWHM remained below 30 ppm, as expected for diamagnetic ^{7}Li or ^{19}F species. Slight chemical shift deviations away from the values in **Table S1** are attributed to local structure distortions/strain, e.g., due to the nearby presence of the DRX phase, while the FWHMs of the diamagnetic resonances depend on the size of the corresponding diamagnetic domain, with large domains resulting in sharp resonances, and small domains (≈10 Å) leading to paramagnetically-broadened resonances due to long-range through-space paramagnetic dipolar interactions. The error in the fits is estimated to be ±4 % based on results from various fits of similar quality.

Supplementary Note 4: Washing procedure for as-synthesized DRX powders

As-synthesized DRX powders were washed using the custom glassware pictured in **Figure S2**. First, a thin filter paper was placed inside the custom glassware on top of a 10 µm glass fritted disc to prevent clogging. An additional layer of filter paper shaped as a cup was placed inside the glassware to prevent sample loss. The apparatus was transferred to an Ar-filled glovebox and around 300 mg of DRX powder sample were loaded into the glassware and sealed with a rubber septum. Outside the glovebox, the loaded glassware was connected to a Schlenk line and the DRX powder was continuously flushed through with N_2 to avoid air and moisture exposure. Outgassed deionized (ODI) water was prepared using the freeze/pump/thaw method (3 cycles) to remove any trace of dissolved O_2 or CO_2 and conserved in a double neck Schlenk flask under N_2 and sealed with a rubber septum. The DRX powder was washed using 5 mL of room temperature ODI water collected from the double-neck Schlenk flask using a syringe and injected through a rubber septum directly on top of the DRX sample. 5 mL of ODI water was enough to cover the DRX powder and was pushed through with N_2 pressure. The N_2 flow was kept on for 24 hours after washing to dry the sample. The washed DRX sample was then further dried in the antechamber of an Ar-filled glovebox under dynamic vacuum overnight. The washed powder was recovered inside the glovebox with an ≈80% yield.

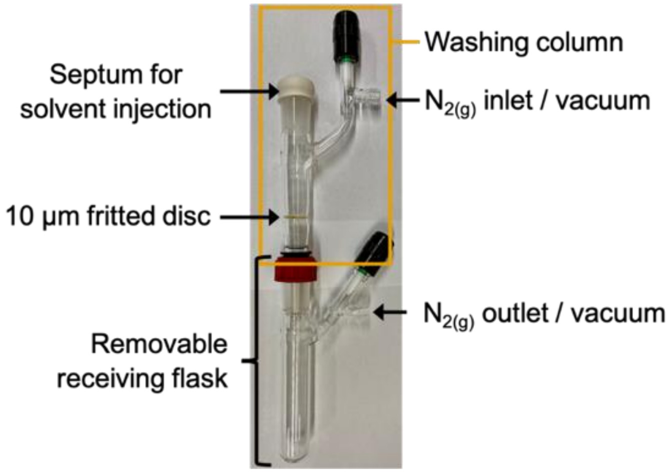

Figure S2. Picture of the custom glassware used to wash as-synthesized DRX powders under an inert atmosphere. The washing column is highlighted by an orange rectangle.

Supplementary Note 5: Determination of the stoichiometry of the DRX phase and overall sample composition

As mentioned in the main text, the overall stoichiometry of the cathode can then be determined if we assume no cation and no anion vacancies in the rock salt structure. The assumption of no cation vacancies is justified by the fact that a rather larger amount of excess Li (10 wt.%) is used in all of our syntheses, which tremendously reduces the probability of forming such vacancies in the rock salt structure (we indeed find residual Li_2CO_3 precursor in our synthesis products, indicating that not all of the excess Li has been volatilized). Besides, Mn and Ti volatility is insignificant at the temperatures used to synthesize DRX compounds, and we find no evidence for Mn- and Ti-containing side products that could reduce the transition metal content in the DRX phase. Hence, it is reasonable to assume that there are no cation vacancies in the DRX phase. Regarding anion vacancies, unlike metallic monoxides, which contain anion (TiO_x, VO_x) vacancies driven by their unique electronic structures, there are few examples of non-metallic lithium transition metal oxides (Li-M-O, M = Mn/Fe/Ni/Co) containing bulk anion vacancies.[14] Anion vacancies lower the coordination number of the transition metals, which is unfavorable for many transition metals with a strong octahedral crystal field stabilization energy (e.g., Mn^{3+}, Mn^{4+}) that tend to form rock salt-type structures based on edge-sharing MO_6 octahedra. Focusing on the Ti and Mn species relevant to this work, a large-scale statistical analysis of the coordination environments of cations in oxides based on ca. 8000 experimentally-observed compounds has found that Ti^{3+} ions (which would be expected to form in the presence of anion vacancies) and Mn^{4+} ions (formed on delithiation) are almost exclusively found in octahedral sites, while the great majority (>90%) of titanates contain octahedrally-coordinated Ti^{4+}.[15] When it comes to Mn^{3+}, it is predominantly found in octahedral sites in oxides (ca. 66%) and is always octahedrally-coordinated in rock salt-type Li-Mn-O compounds. Based on those observations, it is reasonable to assume that the DRX compounds of interest to this work are very unlikely to contain anion vacancies.

It is also safe to assume that Li_2CO_3 is the only plausible carbonate impurity in the as-synthesized DRX samples. Focusing here on the solid-state synthesis procedure employed for DRX, the only carbonate in the precursor mixture is Li_2CO_3. A recent *in situ* heating XRD study has shown that rocksalt-type transition metal oxides (such as MnO used here as a precursor) facilitate the decomposition of Li_2CO_3 at temperatures as low as 450°C, resulting in complete disappearance of the Li_2CO_3 precursor by 700°C.[16] Hence, at the calcination temperatures used here (800°C for 12 hours under Ar), at least stoichiometric amounts of Li_2CO_3 should have reacted with the transition metal oxide precursors. We further note that any transition metal carbonates (e.g., $MnCO_3$) formed upon heating would decompose to an oxide at temperatures in the range of 250-350°C. Hence, the only possibility for transition metal carbonates to form is upon reaction of transition metal oxides with ambient $CO_{2(g)}$ on cooling. This is very unlikely given that the synthesis is carried out under inert atmosphere, limiting environmental $CO_{2(g)}$, and even if a small amount of air was present in the reaction vessel, MnO (the decomposition product of $MnCO_3$) would react with $O_{2(g)}$ much more rapidly than $CO_{2(g)}$. Indeed, we see no evidence for the presence of crystalline transition metal carbonates in our synchrotron XRD results. For all those reasons, the likelihood of transition metal carbonate formation is extremely low.

XRD and elemental analysis of Li-Mn-Ti-O-F samples

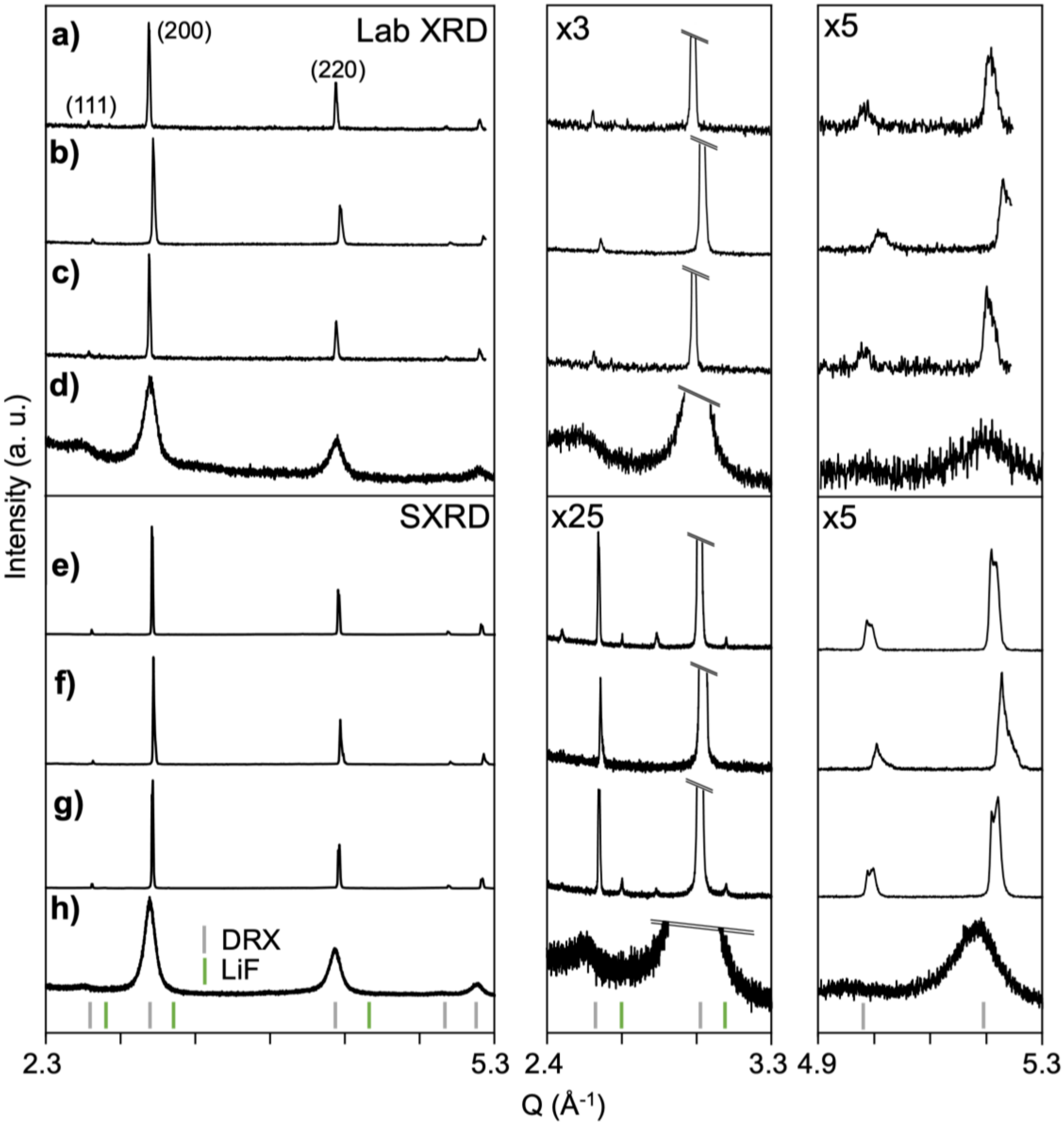

Figure S3. Laboratory and synchrotron XRD patterns collected on **a), e)** as-prepared LMTF25, **b), f)** as-prepared LMTF15, **c), g)** water-washed LMTF25w, and **d), h)** as-prepared LMTF66 powder samples, respectively. The most intense DRX reflections are indexed by their (*hkl*) values. Expected DRX (space group *Fm*-3*m*) reflections for LMTF25 are indicated with grey bars, and LiF reflections are indicated with green bars. The x-axis has been converted from 2θ (°) to Q (Å^{-1}) for simple comparison. The two right panels are expanded views of the 2.4 to 3.3 Å^{-1}, and 4.9 to 5.3 Å^{-1} regions, where the intensities of the reflections were increased highlight weak impurity peaks and peak splitting. Laboratory XRD scans were run for the 10° to 80° 2θ region and are thus terminated slightly earlier than the synchrotron XRD patterns

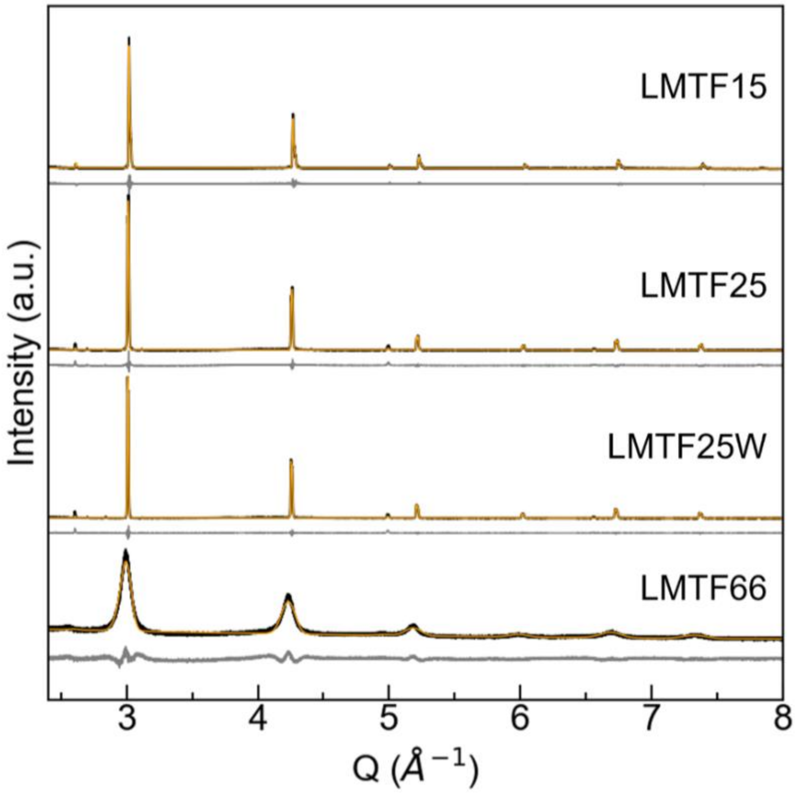

Figure S4. Rietveld refinements of synchrotron XRD patterns for LMTF15, LMTF25, LMTF25W, and LMTF66. Black, yellow, and gray curves correspond to raw data, fit data, and residuals, respectively. Refinements were obtained using TOPAS Academic v7.

Table S3. Lattice parameters obtained from Rietveld refinement of synchrotron XRD patterns collected on the Li-Mn-Ti-O-F powder samples of interest.

Sample	R_{wp}	DRX phase (1)			DRX phase (2)			Quantification		
		a (Å)	vol (Å^3)	R_{Bragg}	a (Å)	vol (Å^3)	R_{Bragg}	DRX (1)	DRX (2)	LiF
LMTF25	11.966	4.171(2)	72.564	2.438	4.178(8)	72.930	2.056	88.74%	10.81%	0.45%
LMTF15	8.966	4.153(9)	71.680	2.110	4.164(3)	72.199	1.976	51.82%	48.18%	0.00%
LMTF25w	11.414	4.173(1)	72.668	2.161	4.179(9)	72.982	2.704	78.75%	21.07%	0.18%
LMTF66	9.388	4.208(2)	74.512	1.736	N/A	N/A	N/A	100.0%	0%	0.00%

Table S4. Li:Mn:Ti:F elemental ratio obtained from ICP-OES and F-ISE on the Li-Mn-Ti-O-F powder samples of interest. Values are normalized to Mn. Error bars are given in parenthesis for all elements except from Mn, and based on the measured deviation from a quality control sample.

Sample	Li (±1%)	Mn (ref)	Ti (±<1%)	F (±5%)
LMTF25	1.31	0.25	0.50	0.17
LMTF15	1.30	0.20	0.53	0.13
LMTF25w	1.28	0.25	0.50	0.12
LMTF66	1.38	0.33	0.32	0.64

Solid-state NMR analysis of Li-Mn-Ti-O-F samples

All spectra shown below were acquired at 2.35 T and 60 kHz MAS (spinning sidebands are denoted with asterisks) using recycle delays of up to 20 s and 5 s for ^{7}Li and ^{19}F, respectively, optimized to ensure complete signal relaxation between scans. The recycle delays used for each samples are indicated in the figure caption. For each spectrum, the overall fit is shown in purple, and an enlarged version of the isotropic region is shown as an inset with individual components obtained from the deconvolution shown in various colors as indicated.

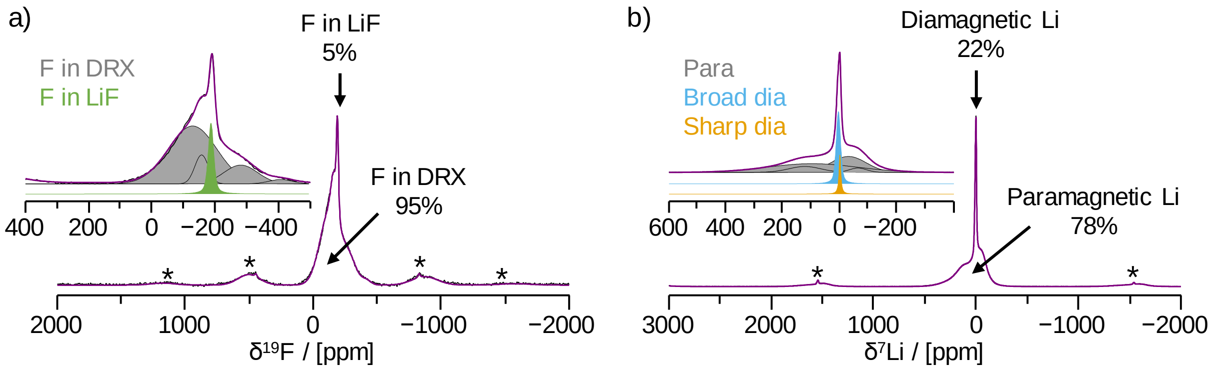

Figure S5. a) ^{19}F and **b)** ^{7}Li spin echo ss-NMR spectra recorded on as-prepared LMTF15 powder using recycle delays of 80 ms and 50 ms, respectively.

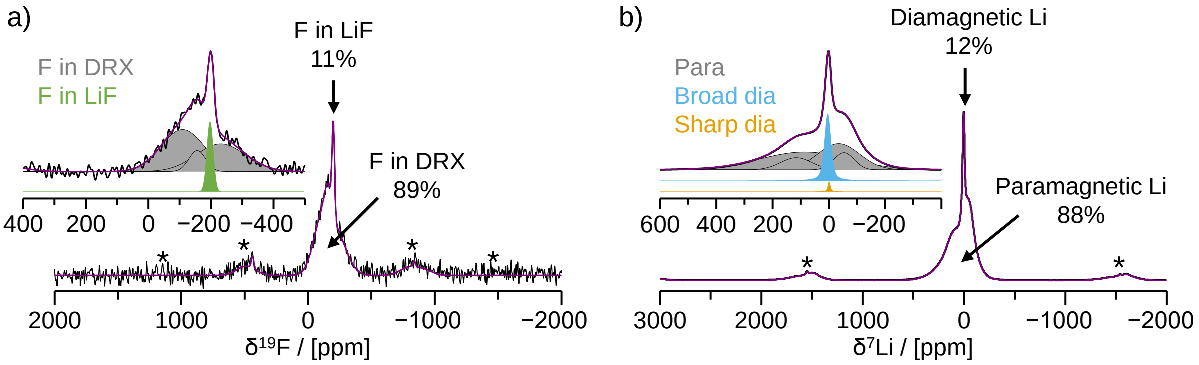

Figure S6. a) ^{19}F and **b)** ^{7}Li spin echo ss-NMR spectra recorded on water-washed LMTF25w powder using recycle delays of 5 s and 50 ms, respectively.

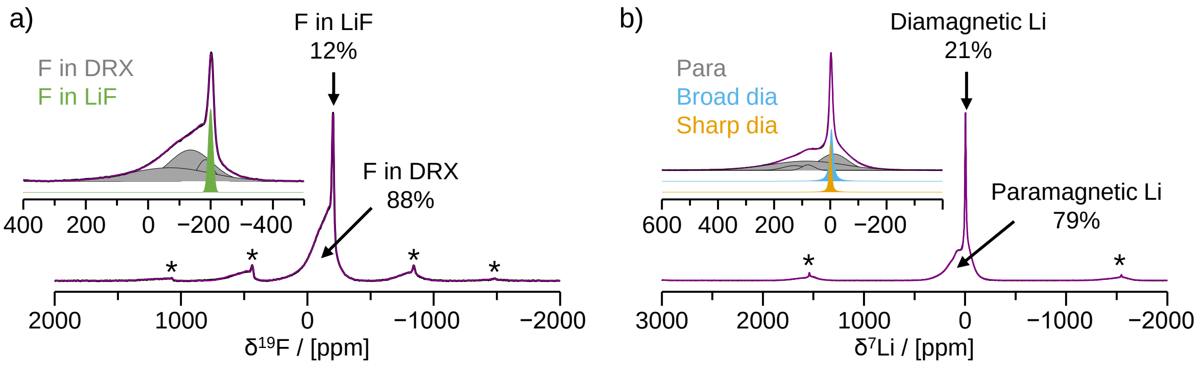

Figure S7. a) ^{19}F and **b)** ^{7}Li spin echo ss-NMR spectra recorded on as-prepared LMTF66 powder using recycle delays of 50 ms and 80 ms, respectively.

Table S5. Distribution of paramagnetic and diamagnetic ^{7}Li and ^{19}F environments in the as-synthesized and water-washed samples of interest, obtained from a deconvolution of the fully relaxed ^{7}Li and ^{19}F spin echo spectra shown above. The error in the fits is estimated to be ± 4 % based on results from various fits of similar quality. More details on the procedure used to fit the ss-NMR spectra can be found in **Supplementary Note 2**. The minimum F stoichiometry in the DRX phase is obtained by combining F-ISE and ^{19}F ss-NMR results.

Sample	Paramagnetic Li (%)	Diamagnetic Li (%)	Min. F in DRX (%) and stoichiometry	Max. F in LiF (%)
LMTF25	82	18	72, $F_{0.12}$	28
LMTF15	78	22	95, $F_{0.12}$	5
LMTF25w	88	12	89, $F_{0.11}$	11
LMTF66	79	21	88, $F_{0.56}$	12

Table S6. Estimated fraction of Li in the DRX phase and in Li-containing diamagnetic impurities (LiF, Li_2CO_3, Li_2O, and/or LiOH) in the as-synthesized and water-washed samples of interest. The fraction of Li in diamagnetic (Mn-poor) environments in the DRX phase is obtained from the probabilities listed in **Table S2** for a random distribution of cations in the structure. The error in the fits is estimated to be ± 4 % based on results from various fits of similar quality. All values recorded here are percentages of the *total* Li content in the sample.

Sample	Para Li in DRX (%)	Dia Li in random DRX structure (%)	Total Li in DRX (%)	Li in Dia impurities (%)
LMTF25	82	8	90	10
LMTF15	78	15	92	8
LMTF25w	88	9	97	3
LMTF66	79	4	82	18

Table S7. Normalized Mn stoichiometry (S_{Mn}) obtained from ICP; probability of forming F environments with no nearest-neighbor Mn (all 6 M1 species in **Figure S1b** are Li or Ti) in the DRX phase, P(F, M1≠Mn), assuming a random distribution of cations; and upper (lower) bound for the F content in the DRX (LiF) phase. The maximum F stoichiometry in the DRX phase is derived from by combining those results with F-ISE results.

Sample	S_{Mn}	P(F, M1≠Mn)	Max. F in DRX (%) and stoichiometry	Min. F in LiF (%)
LMTF25	0.26	0.44	86, $F_{0.15}$	14
LMTF15	0.21	0.52	97, $F_{0.13}$	3
LMTF25w	0.25	0.45	95, $F_{0.11}$	5
LMTF66	0.37	0.29	96, $F_{0.62}$	4

Carbonate titration analysis of Li-Mn-Ti-O-F samples

Table S8. Molar amount of Li_2CO_3 (per mole of DRX) present in as-prepared LMTF25, LMTF15, and LMTF66 powder samples, and in a water-washed LMTF25w powder sample, obtained from carbonate titration. The molecular weight used for each DRX phase is based on the theoretical DRX stoichiometry.

Sample	mmol of Li_2CO_3/mol DRX
LMTF25	0.6 ± 0.5
LMTF15	5.6 ± 0.5
LMTF25w	0.3 ± 0.5
LMTF66	4.4 ± 0.5

References

(1) Sawant, R. M.; Mahajan, M. A.; Verma, P.; Shah, D.; Thakur, U. K.; Ramakumar, K. L.; Venugopal, V. Fluoride Determination in Various Matrices Relevant to Nuclear Industry: A Review. *Radiochim Acta* **2007**, *95* (10). https://doi.org/https://doi.org/10.1524/ract.2007.95.10.585.

(2) Grey, C. P.; Dupré, N. NMR Studies of Cathode Materials for Lithium-Ion Rechargeable Batteries. *Chem Rev* **2004**, *104* (10), 4493–4512. https://doi.org/10.1021/cr020734p.

(3) Peng, L.; Clément, R. J.; Lin, M.; Yang, Y. NMR Principles of Paramagnetic Materials. In *NMR and MRI of Electrochemical Energy Storage Materials and Devices*; The Royal Society of Chemistry, 2021; pp 1–70. https://doi.org/10.1039/9781839160097-00001.

(4) Carlier, D.; Ménétrier, M.; Grey, C. P.; Delmas, C.; Ceder, G. Understanding the NMR Shifts in Paramagnetic Transition Metal Oxides Using Density Functional Theory Calculations. *Phys Rev B* **2003**, *67* (17), 174103. https://doi.org/10.1103/PhysRevB.67.174103.

(5) Middlemiss, D. S.; Ilott, A. J.; Clément, R. J.; Strobridge, F. C.; Grey, C. P. Density Functional Theory-Based Bond Pathway Decompositions of Hyperfine Shifts: Equipping Solid-State NMR to Characterize Atomic Environments in Paramagnetic Materials. *Chemistry of Materials* **2013**, *25* (9), 1723–1734. https://doi.org/10.1021/cm400201t.

(6) Clément, R. J.; Kitchaev, D.; Lee, J.; Gerbrand Ceder. Short-Range Order and Unusual Modes of Nickel Redox in a Fluorine-Substituted Disordered Rocksalt Oxide Lithium-Ion Cathode. *Chemistry of Materials* **2018**, *30* (19), 6945–6956. https://doi.org/10.1021/acs.chemmater.8b03794.

(7) Ahn, J.; Ha, Y.; Satish, R.; Giovine, R.; Li, L.; Liu, J.; Wang, C.; Clement, R. J.; Kostecki, R.; Yang, W.; Chen, G. Exceptional Cycling Performance Enabled by Local Structural Rearrangements in Disordered Rocksalt Cathodes. *Adv Energy Mater* **2022**, *12* (27), 2200426. https://doi.org/10.1002/aenm.202200426.

(8) Ahn, J.; Giovine, R.; Wu, V. C.; Koirala, K. P.; Wang, C.; Clément, R. J.; Chen, G. Ultrahigh-Capacity Rocksalt Cathodes Enabled by Cycling-Activated Structural Changes. *Adv Energy Mater* **2023**, *13* (23). https://doi.org/10.1002/aenm.202300221.

(9) Ménétrier, M.; Bains, J.; Croguennec, L.; Flambard, A.; Bekaert, E.; Jordy, C.; Biensan, Ph.; Delmas, C. NMR Evidence of LiF Coating Rather than Fluorine Substitution in Li(Ni0.425Mn0.425Co0.15)O2. *J Solid State Chem* **2008**, *181* (12), 3303–3307. https://doi.org/10.1016/j.jssc.2008.09.002.

(10) Meyer, B. M.; Leifer, N.; Sakamoto, S.; Greenbaum, S. G.; Grey, C. P. High Field Multinuclear NMR Investigation of the SEI Layer in Lithium Rechargeable Batteries. *Electrochemical and Solid-State Letters* **2005**, *8* (3), A145. https://doi.org/10.1149/1.1854117.

(11) Richards, W. D.; Dacek, S. T.; Kitchaev, D. A.; Ceder, G. Fluorination of Lithium-Excess Transition Metal Oxide Cathode Materials. *Adv Energy Mater* **2018**, *8* (5), 1701533. https://doi.org/10.1002/aenm.201701533.

(12) Szymanski, N. J.; Zeng, Y.; Bennett, T.; Patil, S.; Keum, J. K.; Self, E. C.; Bai, J.; Cai, Z.; Giovine, R.; Ouyang, B.; Wang, F.; Bartel, C. J.; Clément, R. J.; Tong, W.; Nanda, J.; Ceder, G. Understanding the Fluorination of Disordered Rocksalt Cathodes through Rational Exploration of Synthesis Pathways. *Chemistry of Materials* **2022**, *34* (15), 7015–7028. https://doi.org/10.1021/acs.chemmater.2c01474.

(13) Massiot, D.; Fayon, F.; Capron, M.; King, I.; le Calvé, S.; Alonso, B.; Durand, J. O.; Bujoli, B.; Gan, Z.; Hoatson, G. Modelling One- and Two-Dimensional Solid-State NMR Spectra. *Magnetic Resonance in Chemistry* **2002**, *40* (1), 70–76. https://doi.org/10.1002/mrc.984.

(14) Banus, M. D.; Reed, T. B.; Strauss, A. J. Electrical and Magnetic Properties of TiO and VO. *Phys Rev B* **1972**, *5* (8), 2775–2784. https://doi.org/10.1103/PhysRevB.5.2775.

(15) Waroquiers, D.; Gonze, X.; Rignanese, G.-M.; Welker-Nieuwoudt, C.; Rosowski, F.; Göbel, M.; Schenk, S.; Degelmann, P.; André, R.; Glaum, R.; Hautier, G. Statistical Analysis of Coordination Environments in Oxides. *Chemistry of Materials* **2017**, *29* (19), 8346–8360. https://doi.org/10.1021/acs.chemmater.7b02766.

(16) Hua, W.; Yang, X.; Casati, N. P. M.; Liu, L.; Wang, S.; Baran, V.; Knapp, M.; Ehrenberg, H.; Indris, S. Probing Thermally-Induced Structural Evolution during the Synthesis of Layered Li-, Na-, or K-Containing 3d Transition-Metal Oxides. *eScience* **2022**, *2* (2), 183–191. https://doi.org/10.1016/j.esci.2022.02.007.
